# Additive-free MXene inks and direct printing of micro-supercapacitors

**DOI:** 10.1038/s41467-019-09398-1

**Published:** 2019-04-17

**Authors:** Chuanfang (John) Zhang, Lorcan McKeon, Matthias P. Kremer, Sang-Hoon Park, Oskar Ronan, Andrés Seral‐Ascaso, Sebastian Barwich, Cormac Ó Coileáin, Niall McEvoy, Hannah C. Nerl, Babak Anasori, Jonathan N. Coleman, Yury Gogotsi, Valeria Nicolosi

**Affiliations:** 1 CRANN and AMBER Research Centers, Trinity College Dublin, Dublin 2, Ireland; 2 School of Chemistry, Trinity College Dublin, Dublin 2, Ireland; 3 School of Physics, Trinity College Dublin, Dublin 2, Ireland; 4 I-FORM Advanced Manufacturing Research Centre, Trinity College Dublin, Dublin 2, Ireland; 5 A.J. Drexel Nanomaterials Institute and Department of Materials Science and Engineering, Drexel University, Philadelphia, PA 19104 USA

**Keywords:** Synthesis and processing, Two-dimensional materials

## Abstract

Direct printing of functional inks is critical for applications in diverse areas including electrochemical energy storage, smart electronics and healthcare. However, the available printable ink formulations are far from ideal. Either surfactants/additives are typically involved or the ink concentration is low, which add complexity to the manufacturing and compromises the printing resolution. Here, we demonstrate two types of two-dimensional titanium carbide (Ti_3_C_2_T_*x*_) MXene inks, aqueous and organic in the absence of any additive or binary-solvent systems, for extrusion printing and inkjet printing, respectively. We show examples of all-MXene-printed structures, such as micro-supercapacitors, conductive tracks and ohmic resistors on untreated plastic and paper substrates, with high printing resolution and spatial uniformity. The volumetric capacitance and energy density of the all-MXene-printed micro-supercapacitors are orders of magnitude greater than existing inkjet/extrusion-printed active materials. The versatile direct-ink-printing technique highlights the promise of additive-free MXene inks for scalable fabrication of easy-to-integrate components of printable electronics.

## Abstract (web summary)

Printing functional inks is attractive for applications in electrochemical energy storage and smart electronics, among others. Here the authors report highly concentrated, additive-free, aqueous and organic MXene-based inks that can be used for high-resolution extrusion and inkjet printing.

## Introduction

The recent boom in flexible, portable electronics and internet of things has greatly stimulated the design of advanced, miniaturized energy-storage devices^1–3^. However, manufacturing of micro-supercapacitors (MSCs), in particular those that can achieve high energy density with a long lifetime or energy harvesting at a high rate, remains a significant challenge^4^. While elaborate patterning techniques, such as lithography, spray-masking and laser-scribing can partially resolve the problems^5–7^, the sophisticated processing and inefficient material utilization in these protocols limit the large-scale production of MSCs. In other words, incorporating nanomaterials with excellent charge-storage capability into low-cost manufacturing routes is in high demand.

Direct ink writing of functional materials offers a promising strategy for scalable production of smart electronics with a high degree of pattern and geometry flexibility^8–12^. Compared with conventional manufacturing protocols, direct ink writing techniques, such as inkjet printing and extrusion printing, allow digital and additive patterning, customization, reduction in material waste, scalable and rapid production, and so on^13,14^. An important advance for direct ink writing is the incorporation of functional inks with suitable fluidic properties, in particular surface tension and viscosity^9,12,13^. Substantial progress has been made in the ink writing of various electronic/photonic devices^10,12,15,16^ based on graphene^13–15,17,18^, molybdenum disulfide^12^, black phosphorous^16^, etc^19,20^. However, so far only limited success has been reported in achieving both fine-resolution printing and high-charge-storage MSC performance^8^. In addition, in most printable inks, additives (such as surfactants or secondary solvents) are typically used to tune the concentration/rheological properties of the ink, as well as to improve the conductivity of the printed lines. The additional surfactant removal and thermal annealing steps complicate the device manufacturing process. In other words, the formulation of additive-free inks is of significance for a scalable, low-cost, yet efficient printing process.

MXenes are a family of two-dimensional (2D) carbides and nitrides of transition metals (M), where *X* stands for carbon or nitrogen^21^. The most extensively studied MXene, titanium carbide (Ti_3_C_2_T_*x*_, where T_*x*_ represents the terminated functional groups) possesses a high electronic conductivity up to ~10,000 S cm^−1^ and a TiO_2_-like surface^22^, resulting in ultrahigh volumetric capacitance (~1500 F cm^−3^) in Ti_3_C_2_T_*x*_ hydrogel films and high areal capacitance (~61 mF cm^−2^) in Ti_3_C_2_T_*x*_ MSCs^23–25^. While plenty of printed graphene MSCs have been reported with good areal capacitances, but fairly low volumetric capacitances^15,18,26^, to date, there are just a few reports on inkjet printing of MXenes for sensors and electromagnetic interference shielding^27,28^. The only reported MXene printing was achieved on a thermal HP printer^25^, which limits the deposition of the MXene ink on a blank paper with a single pass, and is thus incompatible with most of micro- or nanofabrication procedures, which typically require multiple passes and/or deposits on curved surfaces. For scaling up production and industrial applications of flexible MXene-based devices, a controllable and scalable piezoelectric printing approach, which is compatible with the commercial manufacturing lines, is needed. To date, all-printed MXene MSCs with fine resolution using direct ink printing techniques have yet to be developed.

The main challenges of realizing precise MXene printing lies either in the MXene ink formulation or the solvent evaporation kinetics, or both. Typically, inkjet printing requires a high ink viscosity within a narrow range (1–20 mPa·s) and a suitable surface tension to ensure a stable jetting of single-droplets^9,14,16,29,30^. In addition, surface tension has to be matched with the surface energy and texture of the substrate to allow good wetting^16^. To overcome the “coffee ring” issue^9,14^, inks are usually mixed with surfactants or polymer stabilizers (e.g., ethyl cellulose)^13,15,31^, and/or exchanged with low-boiling-point solvents (e.g., terpineol^11,14,15^ or ethanol^14^) to rapidly solidify the materials. Electronics printed in this way contain residual surfactants/polymers, which need to be removed either through high-temperature annealing^11,15^, chemical treatment^9,14^ or intense pulsed light treatments^13^. These processes are not compatible with most substrates and are especially impractical for MXenes, which may oxidize upon heating in open air^32,33^. In addition, MXene nanosheets typically suffer from a quick precipitation in low-boiling-point solvents, limiting the formation of concentrated MXene ink for efficient additive manufacturing.

Here, we report formulation and direct printing of additive-free, concentrated MXene inks, with high printing efficiency and spatial uniformity. Two types of Ti_3_C_2_T_*x*_ MXene inks, aqueous and organic, in the absence of any additives, were designed for extrusion printing and inkjet printing, respectively. The all-MXene printed MSCs have exhibited excellent areal capacitance and volumetric capacitance. In addition, the protocols of MXene ink formulations as well as printing are general, i.e., ohmic resistors can be inkjet-printed, suggesting the great potential of this printing platform for scalable manufacturing of next-generation electronics and devices.

## Results

### Solvent selection criteria

**Fig. 1:**
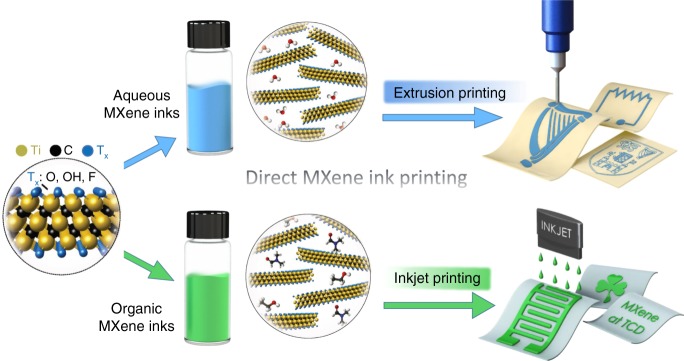
Schematic illustration of direct MXene ink printing. The Ti_3_C_2_T_*x*_ organic inks, i.e., Ti_3_C_2_T_*x*_-ethanol (molecules shown in the bottom panel) are used for inkjet printing of various patterns, such as MSCs, MXene letters, ohmic resistors, etc. The Ti_3_C_2_T_*x*_ aqueous inks (with water molecules shown in the top panel) are designed for extrusion printing of MSCs and other patterns on flexible substrates. As for the MSCs, a gel electrolyte made of H_2_SO_4_-PVA, was coated onto the as-printed patterns and dried naturally, forming all-MXene printed, solid-state MSCs

To minimize the amount of defects on the MXene nanosheets, a less aggressive etching method, so-called minimally intensive layer delamination (MILD), is employed in this research (Supplementary Fig. 1)^34^. The as-obtained multi-layered (m-) Ti_3_C_2_T_*x*_ "cake", which swells after multiple washes (Supplementary Fig. 2), was subjected to vigorous manual shaking in water or bath sonication in organic solvent for delamination. Unlike other 2D materials, which typically require the addition of surfactants or polymer stabilizers^12,13^, the negative electrostatic charge on the hydrophilic Ti_3_C_2_T_*x*_ nanosheets leads to stable aqueous inks containing clean and predominantly single-layered flakes (Supplementary Figs. 3, 4). On the other hand, by selecting suitable organic solvents with a high polarity and a high dispersion interaction strength (dispersion Hansen solubility parameter)^3,35^, stable, concentrated MXene organic inks can be formed. It is worth mentioning that solvents, such as methanol, with a low boiling point typically possess a low polarity index, and so poorly disperse MXene nanosheets and thus limit the dispersion stability. While a binary-solvent system with a low boiling point and low-medium polarity index would allow faster evaporation, the MXene ink concentration and, as a result, the printing resolution and efficiency, could be compromised compared with pure organic solvents with a high boiling point/polarity index. These two types of viscous inks are used for direct ink writing, namely, organic inks for inkjet printing and aqueous inks for extrusion printing, such as MSCs (Fig. 1).

### Formulation of inkjet-printable MXene organic inks

**Fig. 2:**
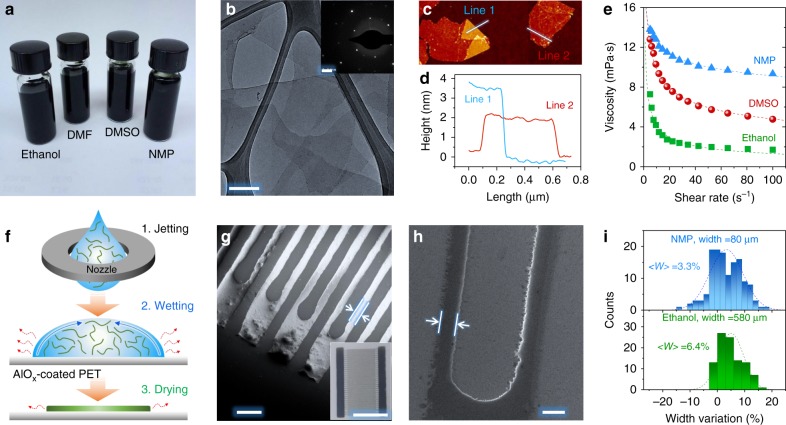
Characterization of MXene organic inks. **a** Photos of various MXene organic inks. **b** TEM image of MXene nanosheets from NMP ink. Inset shows the selected area electron diffraction (SAED) pattern. Scale bar = 200 nm and = 2 1/nm in the inset. **c** AFM image and **d** the corresponding height profiles along the lines in (**c**). **e** Viscosity plotted as a function of shear rates for different MXene organic inks. The data were fitted according to the Ostwald-de Waele power law: $\eta = k\gamma ^{n - 1}$, where *k* and *n* are the consistency and shear-thinning index, respectively^46^. **f** Scheme of fine resolution of inkjet printing of MXene organic inks. The curved green lines represent MXene nanosheets while the arrows indicate the inward (blue) and outward (red) flows of the droplet. Three critical steps, namely, stable jetting, good substrate wetting and droplet drying, control the spatial uniformity of the resultant printed patterns/lines. SEM image of the inkjet-printed MSC using, **g** the NMP ink (inset shows the whole device) and **h** the ethanol ink. Scale bar in (**g**) and (**h**) = 200 µm and = 1 cm in the inset of (**g**). The distances between the two arrows in (**g**) and (**h**) are 50 µm and 130 µm, respectively. **i** width variation of inkjet-printed MXene lines printed using NMP (top) and ethanol (bottom) inks

We start by describing the formulation of inkjet-printable MXene organic inks. Four organic solvents, N-Methyl-2-pyrrolidone (NMP), dimethyl sulfoxide (DMSO), dimethylformamide (DMF) and ethanol that disperse MXene forming stable colloidal solutions^35^ were used to delaminate the nanosheets and give MXene inks (Fig. 2a, Supplementary Figs. 5, 6), as detailed in Methods. Transmission electron microscopy (TEM) images and electron diffraction (ED) of the MXene nanosheets from the ink are shown in Fig. 2b and inset as well as Supplementary Fig. 6b–f. The MXene inks were found to be composed of monolayers to few-layered nanosheets. From the TEM histogram, the flakes in various organic inks possess a mean lateral dimension in the range of ~1–2.1 µm (Supplementary Fig. 7). The atomic force microscopy (AFM) analysis further confirms that the suspended nanosheets are predominantly single-layered (Fig. 2c, d), agreeing with previous reports^24^.

By sealing all the organic inks in Ar-filled hermetic bottles and storing them in a refrigerator^32^, no changes in ink stability (that is, re-aggregation) have been observed over the course of 12 months except in the case of the DMSO ink (Supplementary Fig. 6g–j), which precipitated after 6 months. This necessitates future studies to reveal the possible reasons, as the colloidal stability of the ink is crucial for printing. For instance, measuring the extinction and absorption spectra of DMSO ink over time could provide the decay rate and other insights, however, this is beyond the scope of this work. Nevertheless, the shelf-life of all these inks are over a timeframe that is viable for inkjet printing^16^.

To reach a fine-resolution printing, MXene inks should be designed for stable jetting (that is, no secondary droplet formation after each electrical impulse)^16^. The inverse Ohnesorge number *Z* is commonly used as a figure of merit to predict if an ink will form stable drops: $Z = \sqrt {\gamma \rho D} /\eta$^9^, where *Z* depends on surface tension (*γ*), density (*ρ*), viscosity (*η*) and nozzle diameter (*D*). The viscosity–shear rate plots indicate non-Newtonian characteristics and shear-thinning (pseudoplastic) behaviour in the organic inks (Fig. 2e and Supplementary Fig. 8)^36^. Based on the inks' rheological properties (Supplementary Table 1), the *Z*~2.6 for ethanol ink is slightly higher than those of DMSO (*Z*~2.5) and NMP (*Z*~2.2) inks. The *Z* values of all-MXene organic inks are well within the optimal *Z* value range for stable jetting (1 < *Z* < 14)^12,16^.

After jetting, proper substrate wetting and ink drying are crucial for uniform material deposition. Previous best practices suggested that the ink *γ* should be 7–10 mN m^−1^ lower than the substrate surface energy^16^. While *γ* of ethanol (~22.1 mN m^−1^) well matches that of common substrates like glass (~36 mN m^−1^) and polyethylene terephthalate (PET, ~48 mN m^−1^)^16^, the MXene-ethanol ink concentration is fairly low (0.7 mg mL^−1^). DMF, NMP and DMSO on the other hand, possess high *γ* (~37.1, 40.8 and 43.5 mN m^−1^, respectively), with MXene concentrations up to 12.5 mg mL^−1^, but require additional treatment such as solvent transfer to match the substrate^16^. Here we choose an AlO_x_-coated PET substrate (~66 mN m^−1^)^37^ to solve the substrate wetting issue for all organic inks, as shown in Fig. 2f. The representative inkjet-printed lines using NMP and ethanol (Fig. 2g, h) inks showcase a high printing resolution without undesirable coffee ring effects; the nanosheets can be clearly seen on the smooth surface, forming a conductive film (Supplementary Fig. 9). By inkjet printing the NMP ink, a line (width, gap, spatial uniformity) of (~80 µm, ~50 µm, ~3.3%) was achieved, in contrast to (~580 µm, ~130 µm, 6.4%) in the ethanol-based inks (Fig. 2i and Supplementary Fig. 10). On the other hand, depositing NMP ink onto Kapton and glass substrates led to non-uniform lines (Supplementary Fig. 11), highlighting the importance of substrate selection in achieving high-resolution printing of MXene inks.

### All-MXene inkjet-printed patterns

**Fig. 3:**
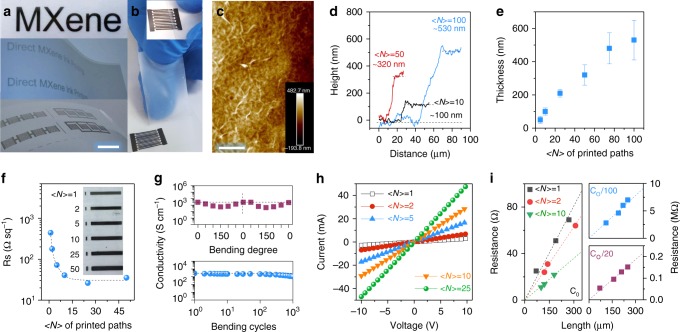
Inkjet printing of MXene organic inks. **a** Optical images of inkjet-printed “MXene” word (top), “Direct MXene Ink Printing” word (middle) and MSCs (bottom) supported on AlO_x_-coated PET. A total of 20 MSCs with different combinations and 80 letters were printed as a demonstration, indicating a high reproducibility of the inkjet printing. Scale bar = 1 cm. **b** Multiple peeling tests of an inkjet-printed MSC (inset) using scotch tape. No material is stuck to the tape after ten peels, indicating a strong adhesion between the printed lines and the AlO_x_-coated PET substrate. **c** AFM image of the inkjet-printed line, showing a homogenous surface consisting of interconnected nanosheets. Scale bar = 10 µm. **d** The height profiles of inkjet-printed lines with different number of paths, <*N*>. **e** The line thickness plotted as a function of <*N*>. The lines in (**d**) and (**e**) were printed using NMP ink with a concentration of 12.5 mg mL^−1^. **f** The sheet resistance, *R*_s_, plotted as a function of <*N*>. Inset shows the optical images of various printed lines (2 cm in length) with different <*N*>. **g** The electronic conductivity changes as a function of bending degree (top) and number of bending cycles (bottom). One cycle is defined as bending the printed line to 150° then releasing to 0° (flat). **h** I–V curves of lines with different <*N*>. **i** Resistance of the printed lines with different <*N*> plotted as a function of length (left). An excellent linearity is observed in all the as-printed lines. The right panel shows resistance of lines with <*N*> = 1 using NMP ink, indicating a line resistivity of 620 kΩ µm^−1^ and 26 MΩ µm^−1^ achieved in the inks diluted by 20 times (~0.63 mg mL^−1^, bottom right) and 100 times (~0.13 mg mL^−1^, top right), respectively

Figure 3a shows examples of inkjet-printed patterns, such as “MXene” word, printed using NMP-based MXene ink on an AlO_x_-coated PET substrate under ambient conditions. In particular, all-MXene MSCs can be produced in series and/or parallel (Fig. 3a). These MSCs are strongly adhered to the substrate, as confirmed by the clean scotch tape observed after peeling the MSC ten times (Fig. 3b, Supplementary Movie 1). These MSCs showcase an interconnected nanosheet network in the film, according to the atomic force microscopy (AFM, Fig. 3c). By adjusting the printing pass, both the thickness (obtained from AFM height profile, Fig. 3d) and sheet resistance (*R*_s_) can be effectively tuned. The line thickness and roughness increase linearly with the number of paths (<*N*>, Fig. 3e and Supplementary Fig. 12). For instance, a line thickness of 100 ± 21.5 nm and 530 ± 120 nm is achieved when <*N*> = 10 and 100, respectively. The *R*_s_ of the printed lines (2-cm-long) quickly decreases from 445 Ω/sq (<*N*> = 1) to 35 Ω/sq (<*N*> = 50), as shown in Fig. 3f and the inset. In addition, the inkjet-printed lines become darker with increasing <*N*>, indicative of uniform printing^28^. No oxide was observed on the as-printed lines while the film surface became rougher in the lines that were exposed to the ambient lab environment for 6 months (Supplementary Fig. 13). This is in good agreement with X-ray photoelectron spectroscopy (XPS) results (Supplementary Fig. 14a), as the deconvoluted Ti 2p core-level spectrum of the printed line produced using NMP ink (<*N*> = 2) is similar to that of fresh MXene^38^, indicating that the pristine nanosheets are preserved after evaporation of the solvent. Although the C=O contribution is negligible in the deconvoluted C 1s core-level spectrum (Supplementary Fig. 14b), the N1s core-level spectrum can be deconvoluted into three peaks, which can be attributed to the trapped and adsorbed NMP molecules in the inkjet-printed MXene lines^39–41^. This result suggests that some NMP molecules residue within the MXene nanosheets after the completion of printing.

The representative printed line (<*N*> = 5) shows an electronic conductivity up to 2770 S cm^−1^ initially and maintains 510 S cm^−1^ after 6 months in ambient air, most probably due to the trapped water among the MXene layers. Moreover, printed MXene patterns are not damaged by substrate deformation, i.e., the conductivity of the as-printed lines can be fully recovered to the initial value upon releasing the bending force (Fig. 3g, top); after 1000 bending cycles, the conductivity decreases to 1093 S cm^−1^ (Fig. 3g, bottom), probably due to the mismatch of the nanosheets (Supplementary Fig. 15).

In printed electronics, printing homogeneous lines is of great importance^14^. Here the reliability and reproducibility of inkjet printing technology allows us to print smooth, even and straight MXene lines on plastic substrates, and enables us to study their electrical properties. Figure 3h shows the current–voltage profiles of various lines with <*N*> ranging from 1 to 25, demonstrating ohmic characteristics. The resistance of these MXene lines is <*N*> and length dependent, showing an excellent linearity with negligible contact resistance (Fig. 3i, left). The line resistivity was determined to be 0.27, 0.21 and 0.12 Ω µm^−1^ for <*N*> = 1, 2 and 10, respectively. Through diluting the initial ink concentration by a factor of 20 (~0.63 mg mL^−1^) and 100 (~0.13 mg mL^−1^), the line resistivity sharply increases to 620 kΩ µm^−1^ and 26 MΩ µm^−1^, respectively at <*N*> = 1 (Fig. 3i, right). This indicates that by adjusting ink concentration, line length and thickness, our direct MXene ink writing technique may offer a simple route to print resistors/conductive wires, with resistance values ranging from a few Ω to several MΩ, on flexible substrates, which are essential components of printed analogue circuits^14^.

### Extrusion printing of all-MXene patterns

**Fig. 4:**
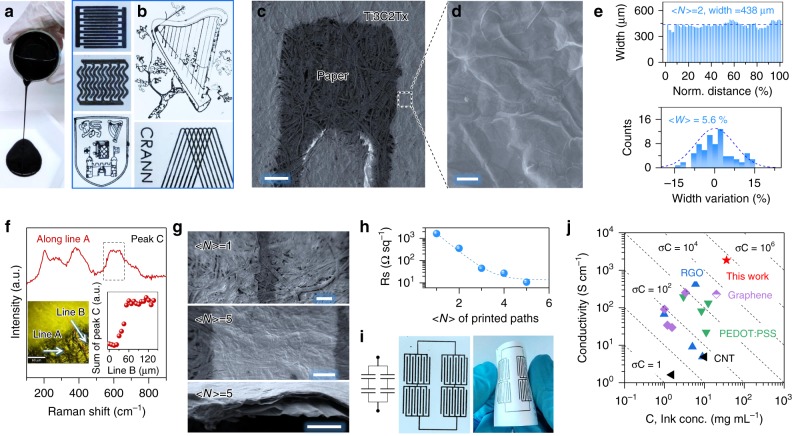
Extrusion printing of MXene viscous aqueous inks. **a** Photo of MXene aqueous ink, showing its viscous nature. **b** Optical images of all-MXene printed patterns, including MSCs with various configurations, on paper. The yield is high using the extrusion printing method, producing > 70 MSCs (<*N*> = 1) on paper based on 1 mL of the MXene ink. **c** Low-magnification SEM image of printed MXene MSC. Scale bar = 200 µm. **d** High-magnification SEM image of MXene MSC of the framed area in (**c**), showing stacked, interconnected MXene nanosheets forming a continuous film. Scale bar = 500 nm. **e** The width distribution and the resultant width spatial uniformity of the extrusion-printed MXene MSCs. **f** Raman spectrum of the extrusion-printed lines along line A, inset shows the sum intensity of peak C along line B. The MXene characteristic peaks are well retained, indicating no oxidation occurred during the extrusion printing. **g** SEM images of printed MSCs with <*N*> = 1 (top) and <*N*> = 5 (middle). Cross-sectional SEM image of MSC with <*N*> = 5 is shown on the bottom, demonstrating a well-stacked, continuous nanostructure. Scale bar = 100 µm (top and middle) and = 1 µm (bottom). **h** The sheet resistance, *R*_s_, plotted as a function of <*N*>, showing an exponential decay. **i** Extrusion-printed tandem devices (two MSCs in serial and two in parallel) on a paper substrate (left panel), showing a great flexibility (right panel). **j** Comparison of the conductivity of the as-printed lines plotted as a function of ink concentration, showing the advantage of our work in printing highly concentrated inks for highly conductive networks. The data in (**j**) come from ref. ^13^ and references within

We further demonstrate the extrusion printing using MXene aqueous ink due to its suitable fluidic properties^36^, including a viscous nature (Fig. 4a), a high MXene ink concentration (~36 mg mL^−1^) and an apparent viscosity of ~0.71 Pa·s (Supplementary Fig. 16). No sedimentation is observed over the course of 12 months in the aqueous ink (sealed in Ar-filled bottles and placed in a refrigerator). Figure 4b shows various fine-printed patterns. For instance, printing 2 paths gives MSCs with line gap ~120 µm (Fig. 4c), width ~438 µm and spatial uniformity within ~5.6% (Fig. 4d). The as-printed lines consist of interconnected nanosheets, forming a continuous metallic network (Fig. 4e). Well-resolved characteristic Raman peaks of MXene are detected when probing the line along different directions (Fig. 4f and insets), showing no signs of oxidation during extrusion printing.

While the thickness of the lines printed on paper cannot be precisely measured due to the rough substrate surface, we found, generally, that a higher <*N*> corresponds to a thicker continuous film (Fig. 4g and Supplementary Fig. 17), forming a well-percolated nanosheet network that provides a much lower sheet resistance. Indeed, increasing <*N*> from 1 to 5 leads to an exponentially-decayed *R*_s_ from 2000 to 10 Ω sq^−1^ of the extrusion-printed lines (Fig. 4h). The metallic conductivity of the compacted printed lines eliminates the need for additional current collectors and conductive agents, while strong adhesion of stacked MXene sheets due to hydrogen bonds between the layers eliminates the need for a polymeric binder, enabling the scalable production of all-MXene-printed MSCs. For instance, high-resolution, flexible MXene-based tandem MSCs can be extrusion-printed and connected in series and parallel, to meet the energy or power requirements (Fig. 4i). In addition to porous paper, the viscous MXene aqueous ink can be extrusion-printed on solid Al foil without any pre-treatment (Supplementary Fig. 18), showing a homogenous surface and a spatial uniformity to within 3.6% (Supplementary Fig. 19).

To achieve efficient printing, the ideal ink formulation should possess both, high concentration (*C*) and high electronic conductivity (*σ*). Thus, a figure of merit, FoM = *σC* (S cm^−1^ · mg mL^−1^), is commonly used to describe the electronic network properties of a printable ink^13^. A higher FoM value is preferable, as it requires less printed paths to obtain similar electrode conductivity. A combination of MXene bulk behaviour and ultrahigh nanosheet concentration results in a record-high FoM (66,996 S cm^−1^ · mg mL^−1^) in this work (Fig. 4j), much higher than those of other printable inks such as graphene (FoM = 6000 S cm^−1^ · mg mL^−1^)^13^. The ultrahigh FoM in the MXene inks enables both high-resolution printing and excellent charge-storage performance in the printed MSCs, as discussed below.

### Charge-storage performance of printed MSCs

**Fig. 5:**
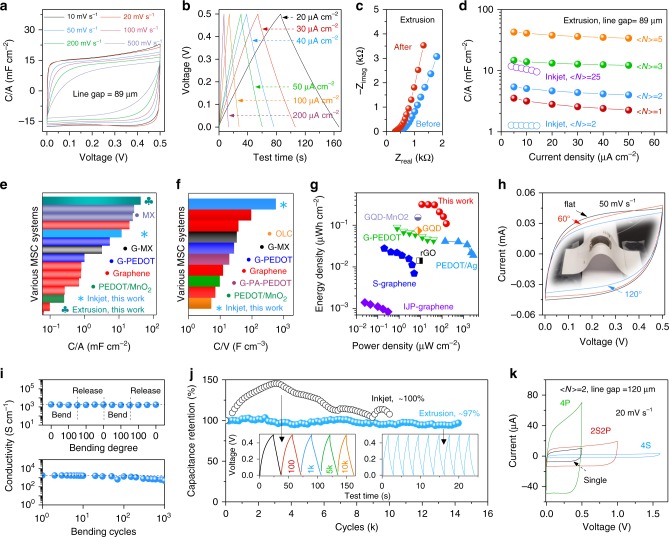
Electrochemical response of inkjet- and extrusion-printed MXene MSCs. **a** Normalized cyclic voltammograms (CV) profiles and **b** galvanostatic charge–discharge (GCD) curves of a typical extrusion-printed MSC (line gap ~89 µm, <*N*> = 3). **c** Electrochemical impedance spectroscopy of extrusion-printed MSC before and after the CV tests at various scan rates. **d** Areal capacitance of inkjet- and extrusion-printed MSCs with different <*N*>. An areal capacitance of 1.3 and 12 mF cm^−2^ is achieved with inkjet printing of <*N*> = 2 and 25, respectively. **e** Areal capacitance (C/A) and **f** volumetric capacitance (C/V) comparison of this work to other reported MSC systems, showing much higher C/V of our printed MXene MSCs than other reports. **g** Ragone plot comparison of this work (extrusion-printed MSC with <*N*> = 5) to other MSC systems. Detailed references and specific values in (**e**)–(**g**) can be found in the Supplementary Information (Supplementary Table 2–4). **h** CVs of extrusion-printed MSC supported on a paper substrate (inset) under different bending degrees. **i** Electronic conductivity of the extrusion-printed lines plotted as a function of bending degree (top) and bending cycles (bottom). **j** Long-term cycling of inkjet- and extrusion-printed MSCs with current densities of 14 and 200 µA cm^−2^, respectively. Insets are the typical GCD curves, showing capacitive behaviour during cycling, indicating that the excellent electrochemical performance is not due to parasitic reactions. **k** Typical CV curves of the as-printed tandem devices, such as printing four MSCs in series and in parallel, and two in series and in parallel. The as-formed tandem devices exhibit capacitive responses, showing the great flexibility of this approach to satisfy different energy/power demands

To demonstrate the possible use of the direct MXene ink printing technique for producing miniature energy-storage devices, the charge-storage performance of both inkjet- and extrusion-printed all-MXene MSCs was evaluated using a sulfuric acid (H_2_SO_4_)-poly(vinyl alcohol, PVA) gel electrolyte^23^. The normalized cyclic voltammetry (CV) and galvanostatic charge–discharge (GCD) curves in Fig. 5a, b as well as the rate response (Supplementary Fig. 20) indicate pseudo-capacitive and high rate behaviour of a typical extrusion-printed MSC (line gap ~89 µm, <*N*> = 3). By optimizing the <*N*> as well as the printed line gap, the electrochemical performance of extrusion-printed MSCs can be changed (Supplementary Fig. 2^1^). In general, increasing the <*N*> results in an enhancement of areal capacitance while minimizing the line gap leads to a substantially reduced time constant, indicative of simple ion diffusion paths (Fig. 5c and Supplementary Fig. 22). For instance, the C/A improves from 3.5 to 43 mF cm^−2^ upon depositing the MXene ink from <*N*> = 1 to 5 (Fig. 5c). Electrochemical impedance spectroscopy (EIS, Fig. 5d) indicates that pseudo-capacitive behaviour is gradually improved upon cycling, agreeing with the CV and GCD results. It is worth noting that the voltage of all-MXene MSCs is limited to 0.5 V due to some parasitic reactions at low rates, highlighting the necessity of printing asymmetric MSCs to enlarge the voltage window.

Similarly, all inkjet-printed MSCs, with a variety of line <*N*> and solvents, demonstrate pseudo-capacitive responses and rate capability up to 1 V s^−1^ (Supplementary Figs. 23–25). In particular, the MSC printed using NMP ink exhibits the highest capacitance, reaching 1.3 and 12 mF cm^−2^ when <*N*> = 2 and 25, respectively (Fig. 5c and Supplementary Fig. 25). Importantly, our inkjet- and extrusion-printed all-MXene MSCs have outperformed most other printed MSCs^10,15^ in terms of areal capacitance (C/A) and volumetric capacitance (C/V), which are two practical metrics for evaluating the charge-storage performance (Fig. 5e)^42^. For instance, printed graphene MSCs typically display a volumetric capacitance below 100 F cm^−3 ^^4,5,10,15^, much lower than our inkjet-printed MXene MSC using the NMP ink (562 F cm^−3^, <*N*> = 25, Fig. 5f). The calculated energy density of the extrusion-printed MXene MSC (<*N*> = 5) reaches as high as 0.32 µW h cm^−2^ at a power density of 11.4 µW cm^−2^, and 0.11 µW h cm^−2^ is still maintained at a high power density of 158 µW cm^−2^ (Fig. 5g). The achieved energy density is an order of magnitude higher than that of MSCs based on printed graphene^43^, sprayed graphene/MXene^44^, etc. We note that by optimization, such as minimizing the line gap, increasing the <*N*>, and/or introducing more pseudo-capacitive sites on the MXene nanosheets, the MSC performance could be further enhanced. There are also dozens of other than Ti_3_C_2_T_*x*_ MXenes to choose from ref. ^21^.

Both as-printed MSCs showcase excellent mechanical flexibility (Fig. 5h, i) and electrochemical cycling performance (Fig. 5j), with capacitance retention of ~97% and ~100% in the extrusion and inkjet-printed MSCs, respectively. The excellent mechanical properties of MXene films can be attributed to the high mechanical strength of Ti_3_C_2_T_x_ flakes^45^ and strong adhesion between the flakes. These high-performance, all-printed MXene MSCs can be arbitrarily connected in series or parallel, forming tandem devices which exhibit capacitive responses, to satisfy specific energy/power demands (Fig. 5k and Supplementary Figs. 26, 27).

## Discussion

We demonstrate additive-free MXene inks and direct printing of high-performance, all-MXene micro-supercapacitors with a high resolution. The printed flexible MSCs demonstrate excellent electrochemical performance, including volumetric capacitance up to 562 F cm^−3^ and energy density as high as 0.32 µW h cm^−2^, surpassing all other printed MSCs, to the best of our knowlege. The direct MXene ink printing technique is of fundamental importance to fields beyond energy storage and harvesting, including electronics, circuits, packaging and sensors, where cheaper and easy-to-integrate components are needed. Of equal importance is that the MXene ink formulation can be achieved by means of a scalable, facile and low-cost route. The additive- and binary-solvent-free, low-temperature printing technique suggests new possibilities for applications in smart electronics, sensors, electromagnetic shielding, antennas and other applications.

## Methods

### Preparation of Ti_3_C_2_T_*x*_ aqueous inks

Thirty-five millilitres of DI-water was added to the above mentioned m-Ti_3_C_2_T_*x*_ “cake”, followed by vigorous shaking by hand/vortex machine for 20 min. This process delaminates the m-Ti_3_C_2_T_*x*_ into single- or few-layered nanosheets well dispersed in water. Then, the mixture was centrifuged at 3500 rpm for 30 min. The top 80% supernatant was collected, and further centrifuged at 5000 rpm for 1 h. After decanting the supernatant, which contains relatively small nanosheets and/or impurities, 10 mL of DI-water was added to the sediment for redispersion by vigorous shaking, resulting in Ti_3_C_2_T_*x*_ aqueous inks.

### Preparation of Ti_3_C_2_T_*x*_ organic inks

In this work, various organic inks were prepared using a solvent-transfer strategy. Typically, the as-prepared Ti_3_C_2_T_*x*_ aqueous dispersion was centrifuged at 10,000 rpm for 1 h. After decanting the supernatant, 20 mL of NMP was added to 0.1 g of Ti_3_C_2_T_*x*_ and the dispersion was sonicated for 30 min. A low speed (1500 rpm, 30 min) centrifugation was then employed to separate the well-dispersed flakes from the aggregated platelets. The supernatant was further centrifuged at 5000 rpm for 30 min. After decanting the supernatant, the sediment was re-dispersed in NMP. The ink concentration can be easily controlled by varying the volume of added NMP. DMSO, DMF and ethanol-based inks were prepared following a similar procedure.

### Inkjet printing of micro-supercapacitors and resistors

The Ti_3_C_2_T_*x*_ organic inks, without any additives, were inkjet-printed using a Dimatix DMP2800 Material printer on a variety of substrates, such as AlO_x_-coated PET (NB-TP-3GU100, Mitsubishi Paper Mills Ltd.), glass, Kapton, etc. The printer was fitted with a cartridge (DMC 11610), producing 10 pL droplets with spacing defined by rotating the print head to a pre-defined angle. The nozzle array consists of 16 identical nozzles 21 µm in diameter spaced 254 µm apart. The substrate was placed onto the vacuum plate of the printer. During printing, the substrate was heated up to 60 °C, while 70 °C was maintained at the ink ejection point in the print head. Patterns, resistors and micro-supercapacitors were printed at a droplet spacing of 25 µm on the coated PET substrate and 100 µm on the glass substrate. Micro-supercapacitor devices with a range of film thicknesses were produced by changing the print pass of the print head. Resistors were printed with different paths (ranging from 1 to 25 Ps) using the pristine Ti_3_C_2_T_*x*_–NMP ink as well as NMP inks diluted by 20 and 100 times. Resistance was measured as a function of the channel length of the resistor.

### Extrusion printing of micro-supercapacitors

The Ti_3_C_2_T_*x*_ aqueous inks, without any surfactants, were extrusion-printed on paper substrates using a 3D printer (Voxel8 Inc., USA). The temperature of the substrate was set to 60 °C while the print head was held at room temperature. The ink was loaded inside the print head and squeezed through the 200 µm-wide nozzle and deposited onto the substrate and then quickly solidified. Printing paths were designed by CAD drawings (SolidWorks 2016, Dassault Systèmes) and converted into G-code by a Voxel8’s proprietary tool path generator to command the x-y-z motion of the printer head. Various patterns and micro-supercapacitors were printed with different print pass, line spacing, width, length, etc.

### Materials characterization

The rheological properties of MXene aqueous inks as well as organic inks, were studied on the Anton Paar MCR 301 rheometer. Morphologies and microstructure of the as-printed lines and devices were studied by scanning electron microscopy and Raman spectroscopy. The surface chemistry of MXene was studied using XPS and the composition analysed by energy-dispersive X-ray spectroscopy (EDX). The electrical conductivity and flexibility of the as-printed lines were evaluated using a two-point probe technique. A detailed description of characterization can be found in Supplementary Methods.

### Electrochemical characterization

Both inkjet- and extrusion-printed MSCs were coated with a layer of gel-like polymer electrolyte made of 3 M sulfuric acid (H_2_SO_4_)-Poly(vinyl alcohol, PVA) gel electrolyte, followed by natural drying. The electrochemical performance of the as-printed MXene MSCs was evaluated on a potentiostat (VMP3, BioLogic) in a voltage window of 0.5 V. CV, GCD, long-term cycling, as well as flexibility of the device were assessed. A detailed description of characterization can be found in Supplementary Methods.

## Supplementary information

Supplementary Information

Supplementary Movie 1

Supplementary Movie 2

Supplementary Movie 3

Supplementary Movie 4

Supplementary Movie 5

Supplementary Movie 6

Supplementary Movie 7

Supplementary Data 1

## Data Availability

The datasets generated during and/or analysed during the current study are available from the corresponding author on reasonable request. We also provide a source data file to include all the source data except the images.
