# Supplementary material for: Additive-free MXene inks and direct printing of micro-supercapacitors
Source: Nat Commun. 2019 Apr 17;10:1795. doi: 10.1038/s41467-019-09398-1 (PMC6470171; doi:10.1038/s41467-019-09398-1)
Supplement: Supplementary file 1 — Supplementary Information [file 41467_2019_9398_MOESM1_ESM.pdf]

Supplementary Information for

Additive-free MXene inks and direct printing of micro-supercapacitors

Chuanfang (John) Zhang,[1,2,*] Lorcan McKeon,[1,3] Matthias P. Kremer,[1,2,4] Sang-Hoon Park,[1,2,] Oskar Ronan,[1,2] Andrés Seral-Ascaso,[1,2] Sebastian Barwich,[1,3] Cormac Ó Coileáin,[1,2] Niall McEvoy,[1,2] Hannah C. Nerl,[1,3] Babak Anasori,[5] Jonathan N. Coleman,[1,3,*] Yury Gogotsi[5,*] and Valeria Nicolosi[1,2,4,*]

[1]CRANN and AMBER research centers, Trinity College Dublin, Dublin 2, Ireland. [2]School of Chemistry, Trinity College Dublin, Dublin 2, Ireland. [3]School of Physics, Trinity College Dublin, Dublin 2, Ireland. [4]I-FORM research centre, Trinity College Dublin, Dublin 2, Ireland [5]A. J. Drexel Nanomaterials Institute and Department of Materials Science and Engineering, Drexel University, Philadelphia, PA 19104, USA

*Chuanfang (John) Zhang (zhangjc@tcd.ie); *Jonathan N. Coleman (colemaj@tcd.ie); * Yury Gogotsi (gogotsi@drexel.edu); * Valeria Nicolosi (nicolov@tcd.ie)

Supplementary Figure

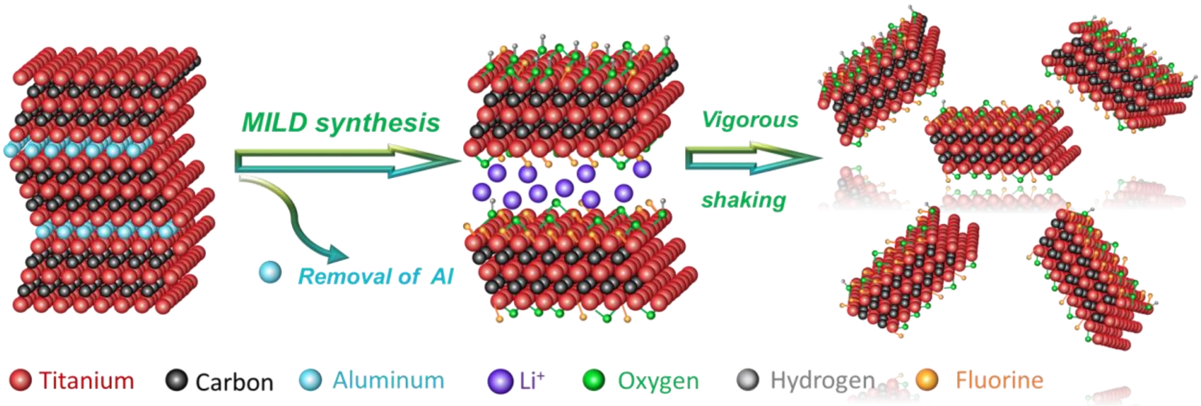

Supplementary Figure 1. Scheme of $Ti_3C_2T_x$ MXene etching and delamination using. A MILD synthesis route to minimize the defects on the nanosheets. After vigorous manual shaking of the swelled MXene aqueous suspension, single- or few-layered MXene was prepared. By sedimentation of the nanosheets, decanting the supernatant, and redispersing the sediment in a small amount of DI-water, concentrated, and viscous MXene aqueous ink was obtained.

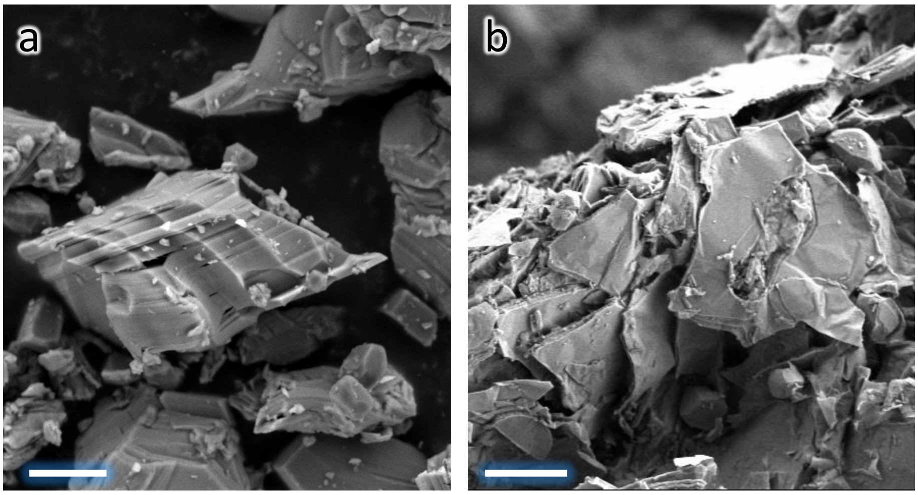

Supplementary Figure 2. Morphological characterization of MAX and MXene. SEM images of **a**, Ti_3AlC_2 MAX phase and **b**, as-obtained m-$Ti_3C_2T_x$ after washing the sediments, showing a certain degree of delamination. This is due to the vigorous shaking during washing, which swells the m-$Ti_3C_2T_x$ through exchanging the pre-intercalated Li^+ with water molecules. Scale bar in a and b = 2 µm.

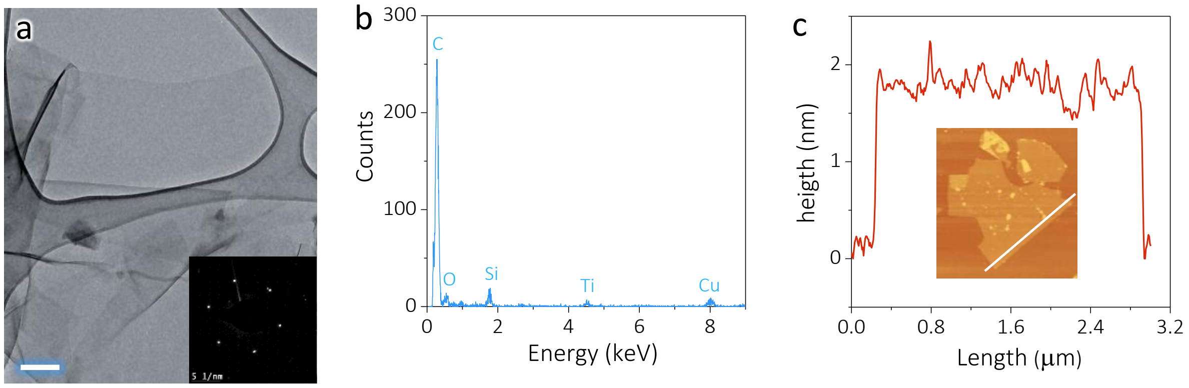

Supplementary Figure 3. Characterization of delaminated $Ti_3C_2T_x$ MXene. **a**, TEM image of the MXene nanosheets with the selected area electron diffraction (SAED) shown in the inset, indicating micron-sized ultra-thin flakes, and confirming that the aqueous ink consists of predominantly single-layer nanosheets. Scale bar = 200 nm. **b**, Energy-dispersive X-ray spectroscopy (EDX) of the nanosheets showed the elemental composition of the ink, confirming that the sample is composed of carbon and titanium. The Cu signal comes from the Cu grids while Si signal may come from contamination. **c**, AFM image and the corresponding height profile of the delaminated nanosheets. The average thickness, ~1.8 nm, suggest the nanosheets are single-layered, agreeing with previous reports.[1,2]

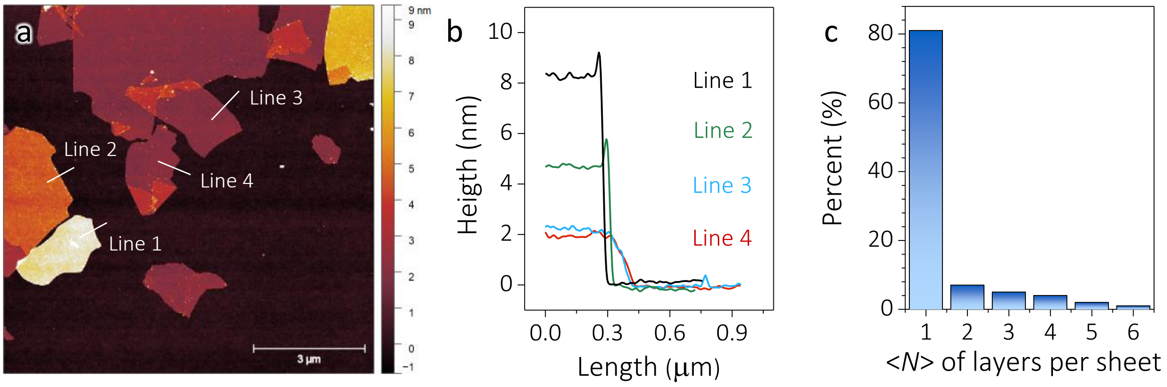

Supplementary Figure 4. AFM statistical analysis number of layers in the MXene aqueous inks. **a**, Representative AFM image of MXene aqueous ink along different lines, **b**, Height profiles along different lines marked in (a), **c**, Histogram of number of layers per sheet with a total of 100 nanosheets were considered. It is clear that the aqueous ink mainly contains single-layer nanosheets, best evidenced by the high content (81%) of single-layer nanosheets (thickness ~1.5 nm).

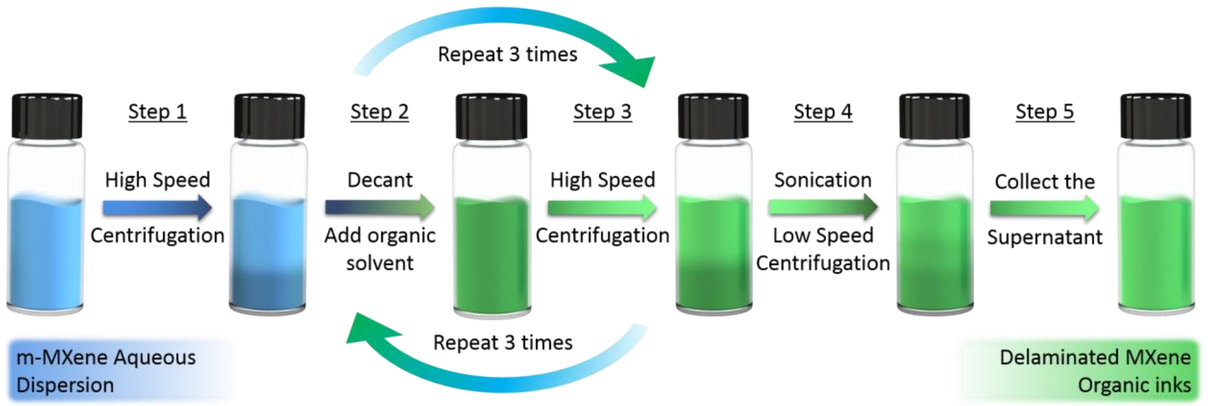

Supplementary Figure 5. Scheme of organic ink preparation. The m-$Ti_3C_2T_x$ sediment was first transferred to an organic solvent, such as NMP. Through direct liquid-phase exfoliation in NMP *via* bath sonication, the m-$Ti_3C_2T_x$ delaminates and disperses in the solvent. To further increase the concentration of the organic dispersion, the as-delaminated nanosheets were sedimented *via* a fast speed centrifugation at 12,000 rpm, followed by redispersing the sediment in a small amount of NMP to reach the desired concentration, forming MXene NMP ink. DMSO, DMF, and ethanol inks were prepared using a similar route.

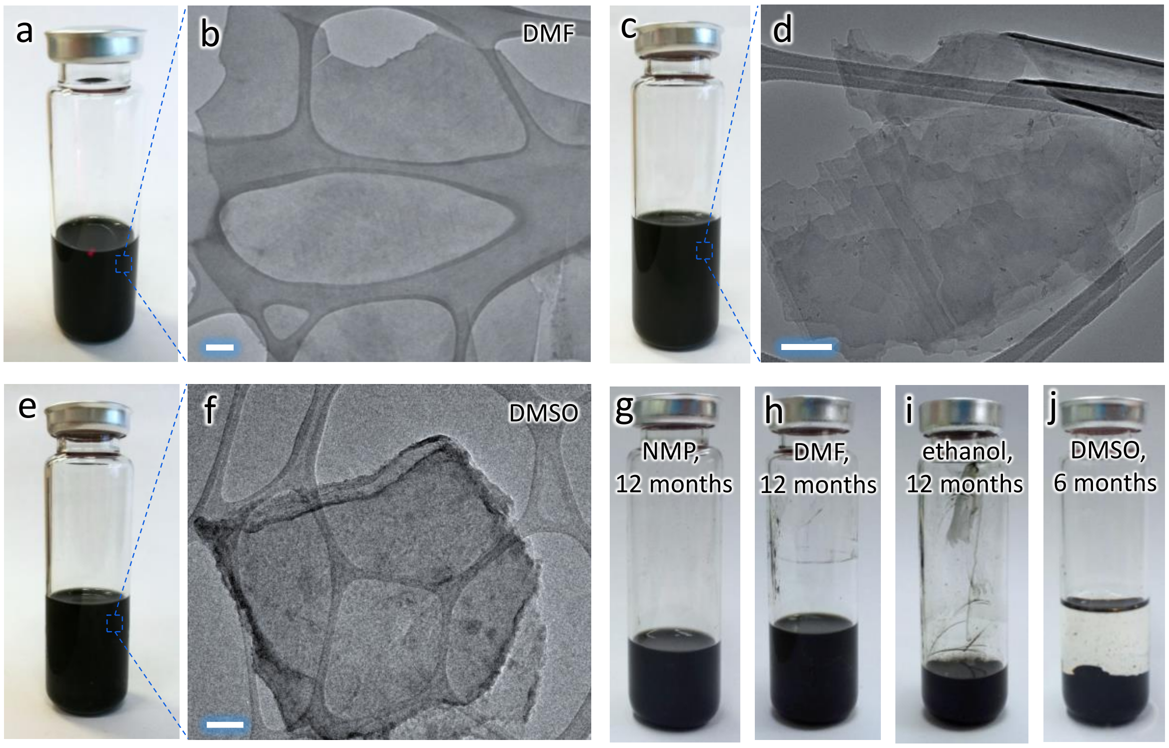

Supplementary Figure 6. Characterization of MXene organic inks. **a**, Optical image and **b**, TEM image of MXene DMF ink. **c**, Optical image and **d**, TEM image of ethanol ink. **e**, Optical image and **f**, TEM image of MXene DMSO ink. **g-j**, Photos of various organic inks after aging for 6 months (for DMSO) and 12 months (NMP, DMF and ethanol). All the organic inks were sealed in the Ar-filled hermetic bottles and stored in a refrigerator. Scale bar in b and f are 200 nm and 100 nm in d.

All inks consist of clean, monolayered nanosheets except DMSO ink where some aggregation phenomena are observed. As a result, clear sedimentation is found in DMSO ink after 6 months, in sharp contrast of the homogeneous ink in NMP, DMF and ethanol inks. Despite that, the stable lifetime of all inks is long enough to be viable for large-scale ink production and inkjet printing.

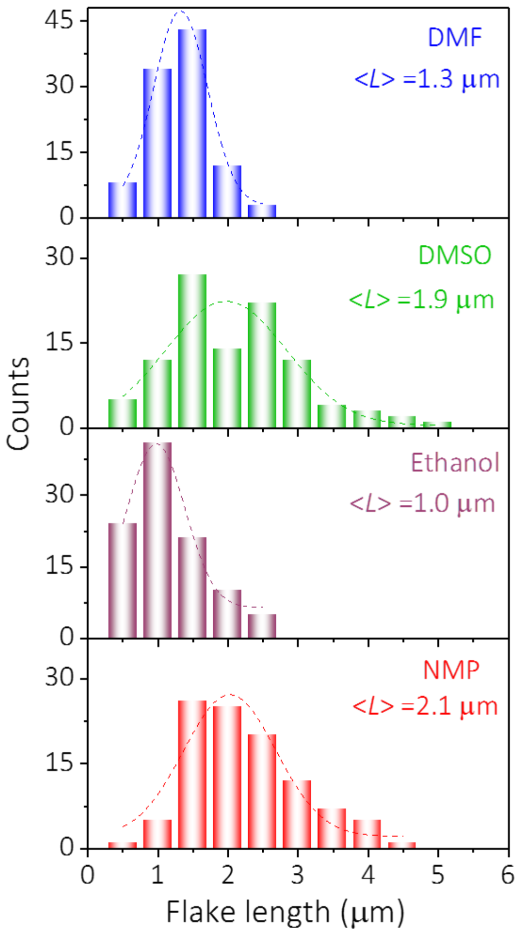

Supplementary Figure 7. $Ti_3C_2T_x$ nanosheet size histogram from TEM statistics of different inks. In general, the mean nanosheet size in the NMP ink is the largest, reaching 2.1 µm, followed by DMSO ink (1.9 µm), DMF ink (1.3 µm) and ethanol ink (1.0 µm).

The lateral size of nanosheets is crucial for inkjet printing; large nanosheets have clogging concerns for the printing nozzle. A general suggestive principle for the printable materials is that the material size should be 1/10 of the nozzle diameter or even lower.

In our case, the diameter of the inkjet printer nozzle is ~21 µm, thus, all of MXene organic inks, which are enriched with predominantly single-layer nanosheets, consist of materials with their size located in the favourable range for the inkjet printing.

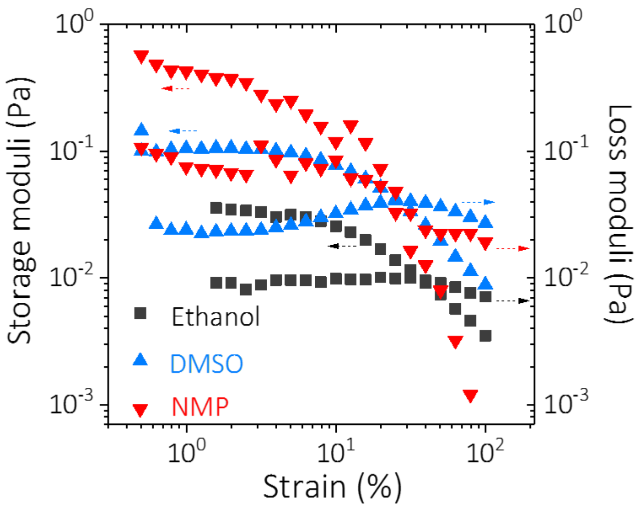

Supplementary Figure 8. Rheological properties of MXene organic inks with the storage and loss moduli plotted as a function of strain. Besides the viscosity measurements, the viscoelastic properties of the inks, in particular, storage and loss moduli, are also important, as these two parameters can be used to investigate regions of linear elastic deformation, yield points, and sample fluidisation, and give insight into the energetics of the sample network.[3] The storage and loss moduli in these viscoelastic materials show much higher storage and loss modulus in NMP ink, followed by DMSO ink and substantially higher than the ethanol ink.

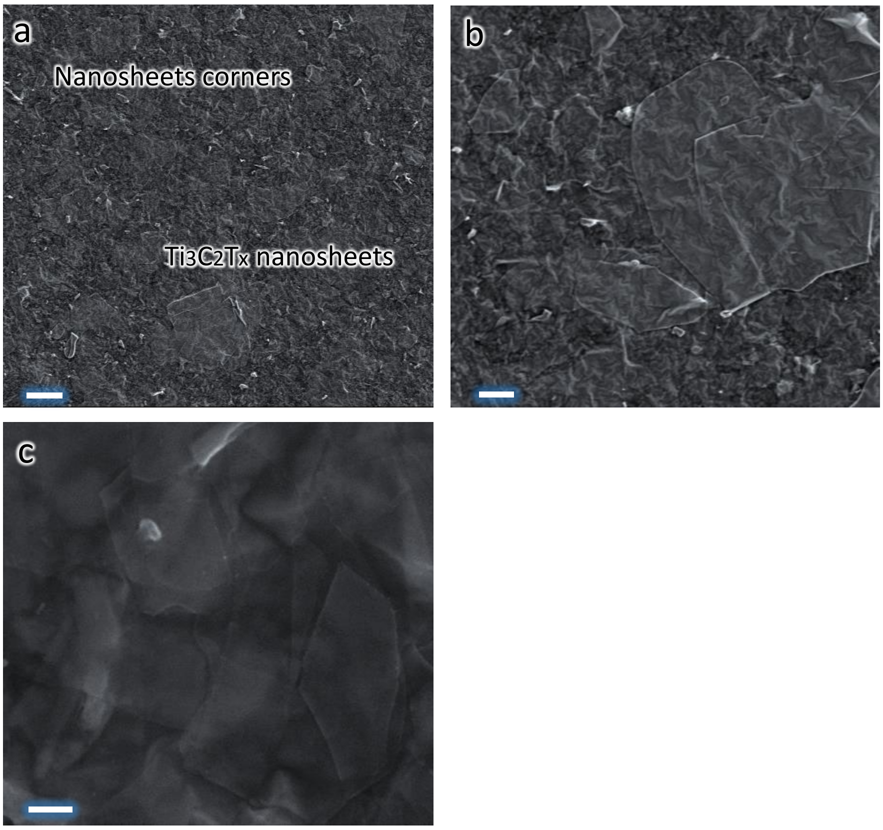

Supplementary Figure 9. Morphological characterization of inkjet-printed lines. **a** and **b**, SEM images of printed lines using NMP ink at different magnifications. A homogeneous surface with a low roughness is observed. In addition, the MXene nanosheets are compactly stacked, forming a continuous film network. **c**, SEM image of printed lines using ethanol ink. Clear MXene nanosheets with lateral size of ~400 nm are found. In addition, these nanosheets have smooth surface without apparent oxidation, indicating the inkjet printing process did not introduce any noticeable damage to the nanosheets. This is important, as the MXene nanosheets perform as the conductive binder, active material as well as current collector simultaneously for MSCs. Scale bars are 2 µm, 1 µm and 100 nm in a, b, c, respectively.

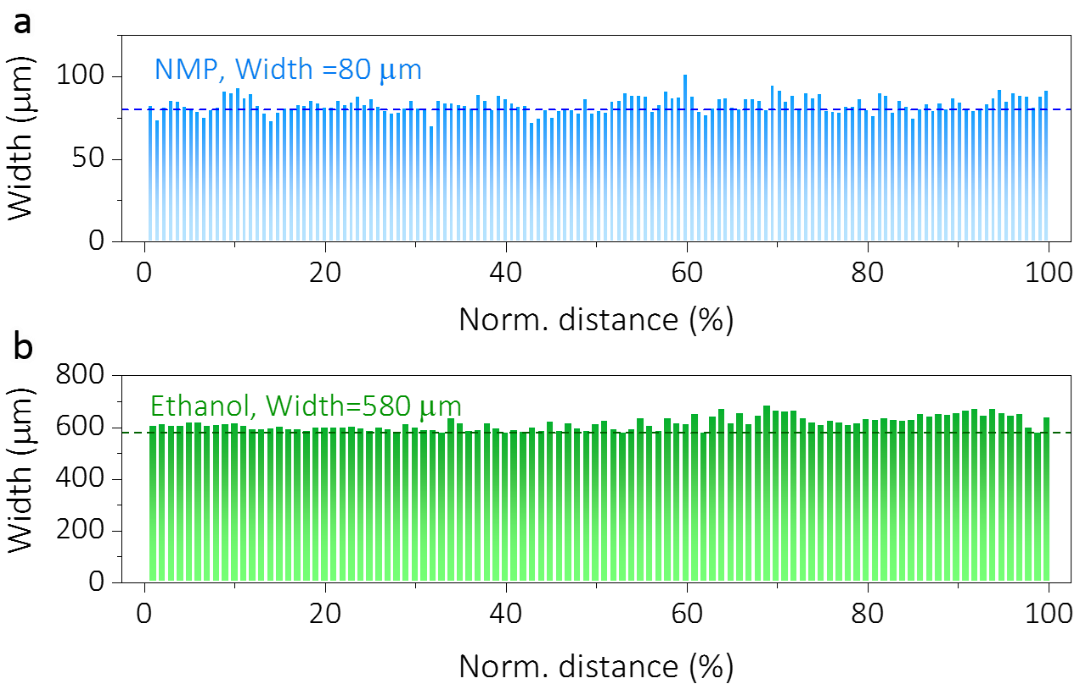

Supplementary Figure 10. Width distribution of as-printed MXene lines. Width distribution along different printed lines using **a**, NMP ink and **b**, ethanol ink.

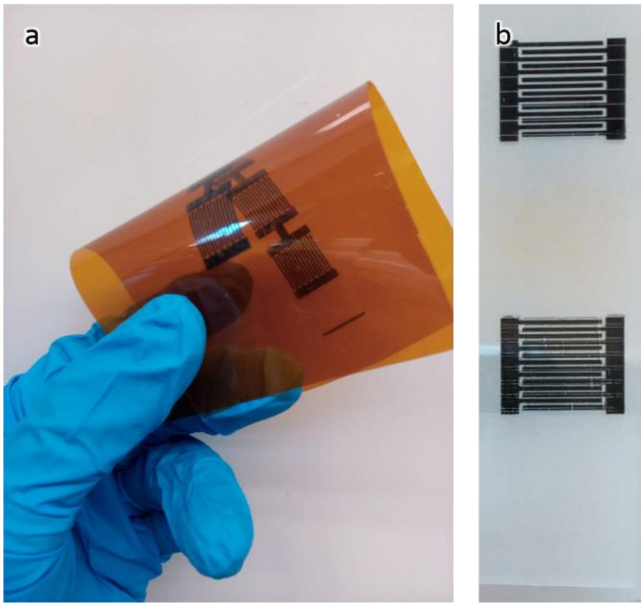

Supplementary Figure 11. Optical images of as-printed MSCs. Inkjet-printed MSCs on **a**, Kapton and **b**, glass substrates. Close examination reveals that printing MXene organic inks on these two substrates are inhomogeneous, best evidenced by the apparent gaps on the straight lines. This is due to the mismatch between the surface energy of the substrate and the surface tension of the ink, resulting in substrate wetting issues. This further highlights the importance of ink formulation and substrate selection in the high-resolution printing of MXene inks.

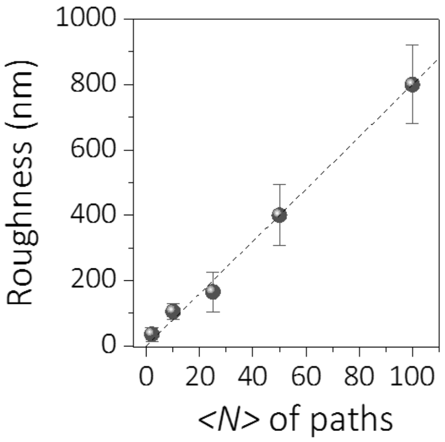

Supplementary Figure 12. The roughness of the inkjet-printed lines plotted as a function of <*N*>. Increasing the number of printing paths results in lines with larger roughnesses.

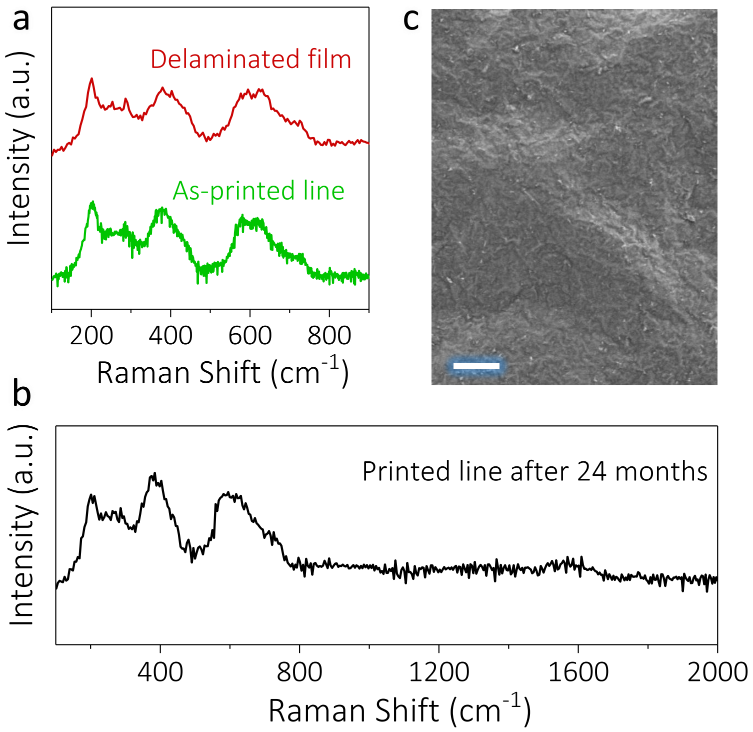

Supplementary Figure 13. Stability of as-printed MXene lines. a and b, Raman spectra of the fresh delaminated film made by vacuum-assisted filtration, inkjet-printed patterns as well as the printed line that was aged under ambient conditions for 24 months. c, SEM image of the printed, aged line (for 24 months). Scale bar is 1 µm in c. The almost identical Raman spectra suggest that the as-printed pattern is stable enough in air, as no oxide peaks are found in the printed line that was exposed in air for 24 months. This agrees with our previous report on the MXene stability, as no oxidation was found in the filtrated films because the dissolved oxygen was expelled from the solution.[4]

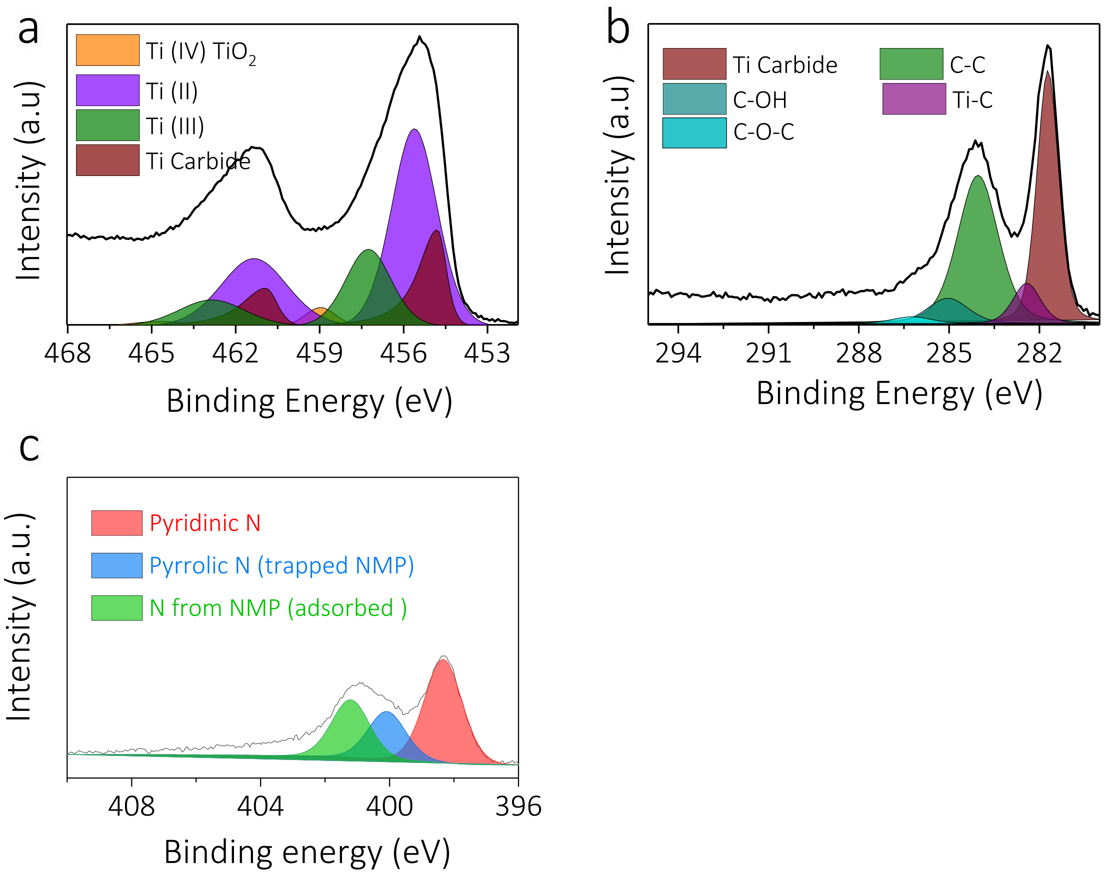

Supplementary Figure 14. Study of solvent residue in the as-printed MXene line. XPS analysis of the $Ti_3C_2T_x$ MXene line inkjet-printed using the NMP ink (<*N*>=2). **a**, the deconvoluted Ti 2p core-level spectrum, **b**, the deconvoluted C 1s core-level spectrum and **c**, the deconvoluted N1s core-level spectrum.

As seen from the Supplementary Figure S14c, there is a certain amount of nitrogen signals in the as-inkjet-printed MXene lines after drying. The N1s core-level spectrum can be deconvoluted into three peaks. The first one centers at 398.5 eV which can be ascribed to pyridinic N. The second one centers at 400.2 eV can be assigned to pyrrolic N (i.e., N coordinated as in the pyrrole molecule), consistent with the presence of NMP molecules that were likely trapped between the MXene nanosheets.[5] The third peak centers at 401.5 eV could probably be explained by the N of the NMP molecules adsorbed onto the MXene nanosheets, when the droplet adsorbed onto an electron acceptor substrate (AlO_x coated PET).[6,7] Conclusively, certain amount of NMP residual has been found in the as-printed MXene line.

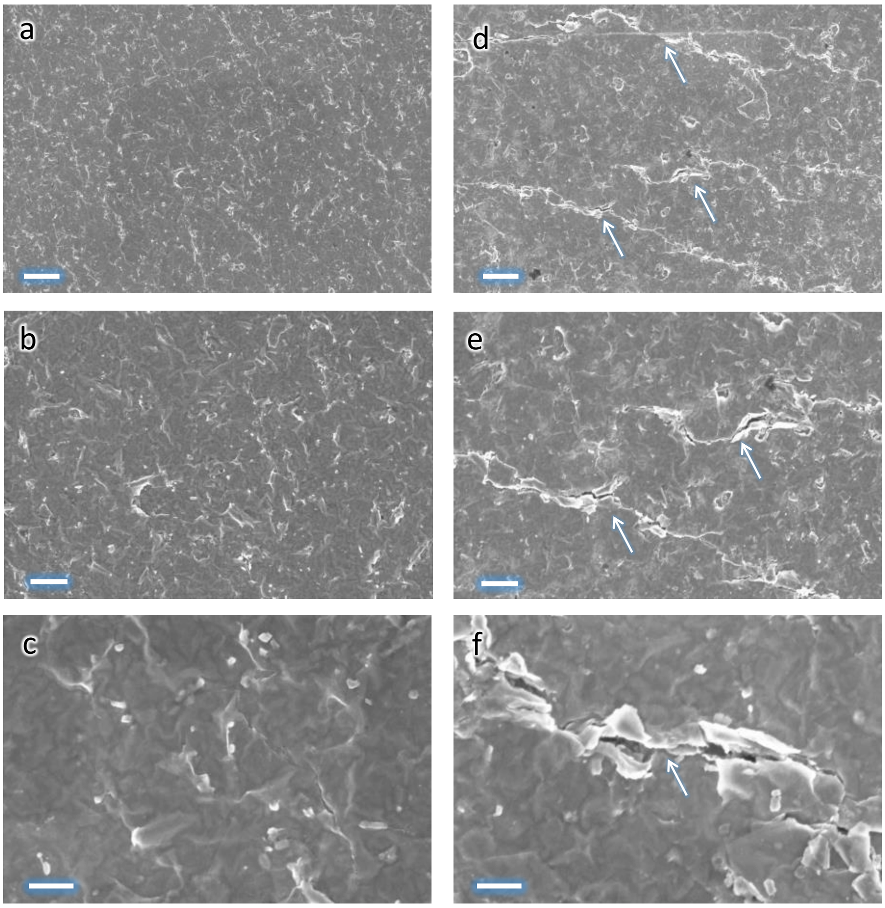

Supplementary Figure 15. Morphological study of as-printed MXene lines upon bending. SEM images of the as-printed MXene line, **a**-**c**, before bending and **d**-**f**, after bending for 1000 cycles. After bending, the printed network has microcracks in (d-f), which may be the cause for the dropped conductivity (Fig. 2g). Scale bars are 2 µm in (a,d), 1 µm in (b,e) and 400 nm in (c,f).

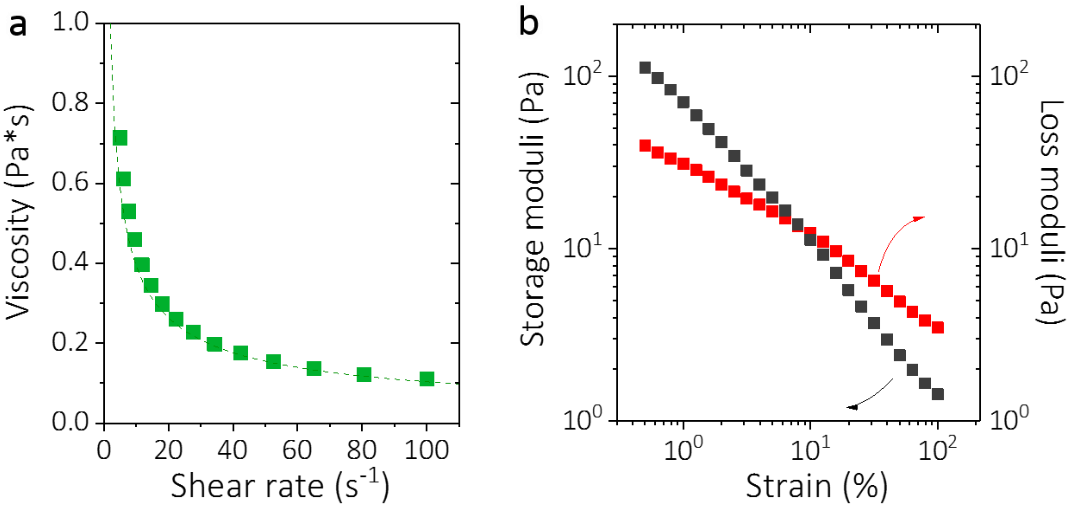

Supplementary Figure 16. Rheological study of viscous MXene aqueous inks. **a**, Viscosity of MXene aqueous ink plotted as a function of shear rate. **b**, Storage and loss moduli of MXene aqueous ink plotted as a function of shear rate.

From the viscosity-shear rate plots in Figure S16a, an apparent viscosity of 0.71 Pa·s coupled with non-Newtonian characteristics and shear-thinning (pseudoplastic) behaviour in the

MXene aqueous ink, are revealed. The viscosity is lower than that of MXene organic inks (Fig. 1e).

The viscoelastic properties of the MXene aqueous ink, namely, storage and loss moduli, are important, as they provide useful information on the linear elastic deformation, yield points, sample fluidisation, etc. As shown in Figure S16b, the storage moduli in the MXene aqueous ink reaches as high as 112 Pa with a loss moduli of 39.8 Pa at a strain of 0.5%. Increasing the strain to 100% leads to a sharp decrease in storage modulus (1.43 Pa) as well as loss modulus (3.49 Pa).

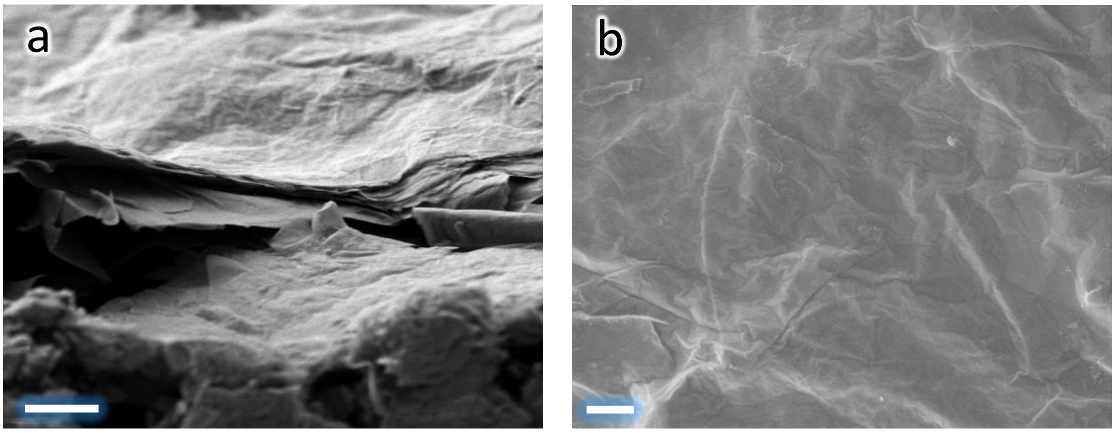

Supplementary Figure 17. Morphological study of as-printed MXene lines. SEM images of **a**, cross-section, and **b**, surface of an extrusion-printed MSC, showing a continuous film supported on the paper. Scale bars are 1 µm in a and b.

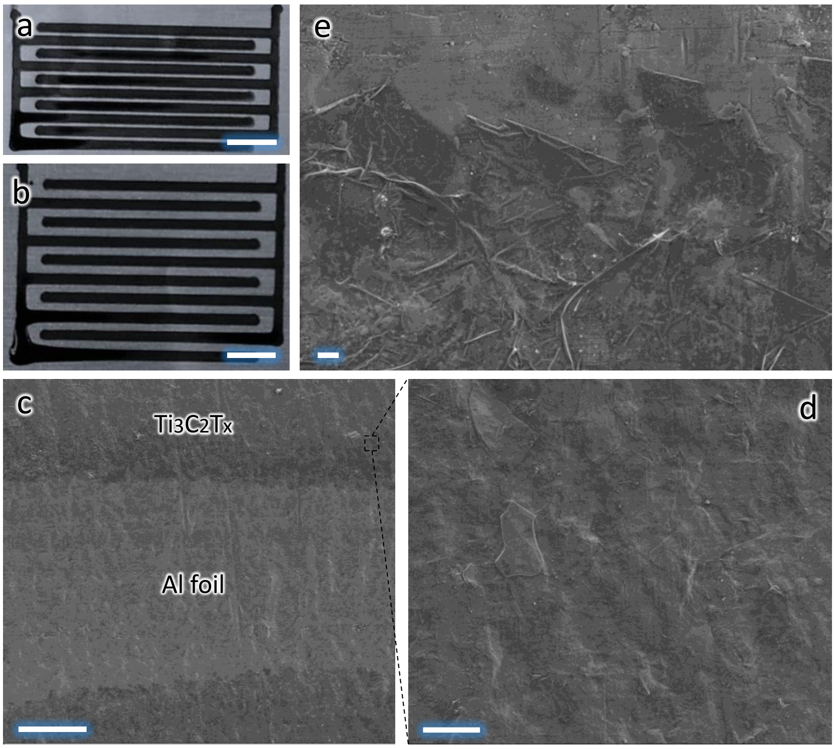

Supplementary Figure 18. Morphological study of as-printed MXene lines on Al substrate. Optical images of extrusion-printed MSCs on Al foil with **a**, smaller line gap (~90 µm) and **b**, larger line gap (~320 µm). Scale bar is 2 mm. **c**-**e**, SEM images of the MSCs at different magnifications, showing a smooth surface with MXene nanosheets clearly seen. It is worth noting that no prior treatment of the Al foil is required, highlighting the versatility of extrusion printing of MXene aqueous ink. Scale bars are 20 µm in c, 4 µm in d, and 500 nm in e.

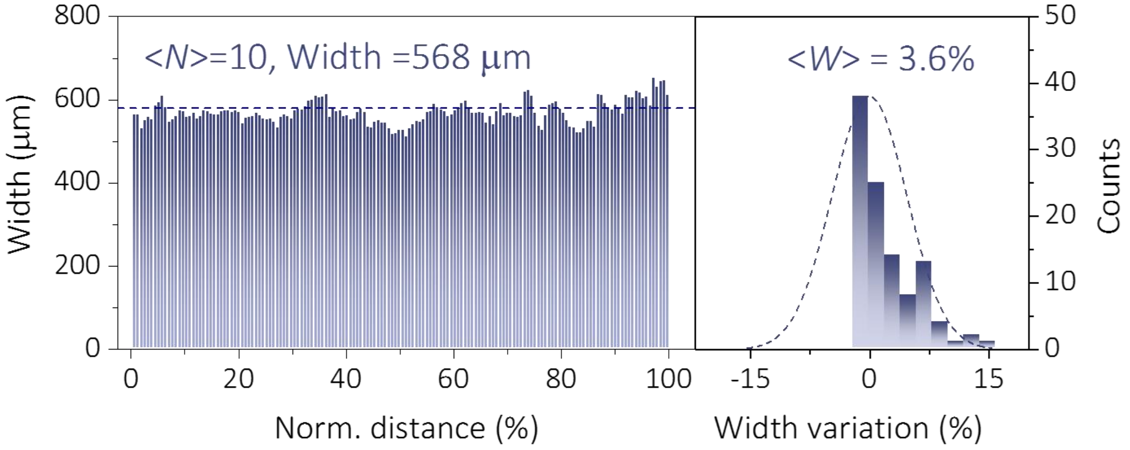

Supplementary Figure 19. Width distribution and spatial uniformity of extrusion-printed MSCs on Al foil. The narrow width distribution indicates a high printing resolution on Al substrate, corresponding to a spatial uniformity <*W*> of 3.6%.

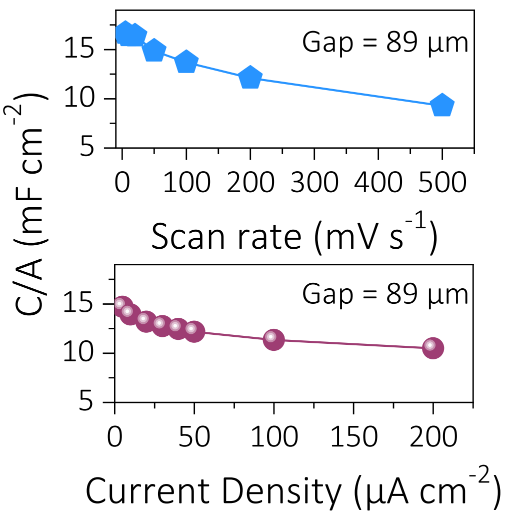

Supplementary Figure 20. Areal capacitance comparison. Areal capacitance calculated from CV curves and GCD profiles of the extrusion-printed MXene MSCs, showing similar values.

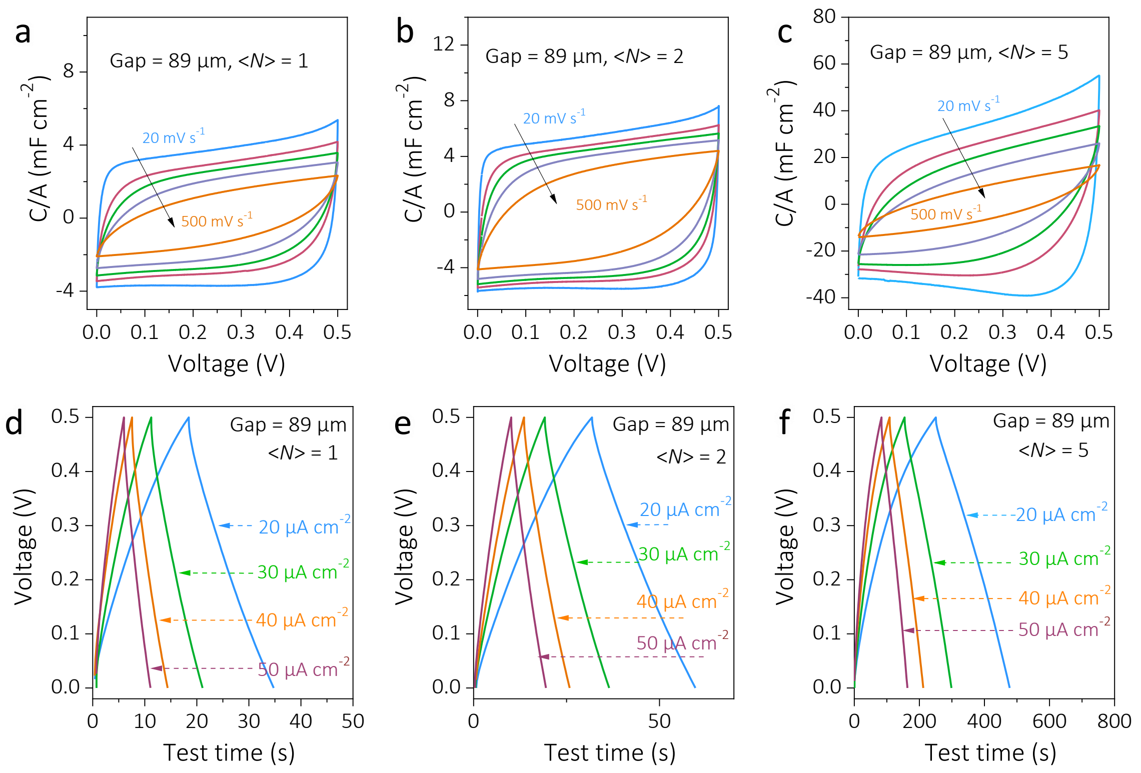

Supplementary Figure 21. Electrochemical characterization of as-printed MXene MSCs. **a**-**c**, CV curves of the extrusion-printed MXene MSCs with different <*N*>. The scan rate varies from 10 mV s^{-1} to 500 mV s^{-1}. All MSCs showcase capacitive behaviour at low scan rates (<100 mV s^{-1}) but gradually become more resistive as the scan rate is beyond 100 mV s^{-1}, due to the limitation of ion diffusion kinetics. **d**-**f**, The corresponding GCD profiles of the extrusion-printed MXene MSCs with different <*N*> at various current densities. The curves are not symmetric at low rates, suggesting redox reactions.

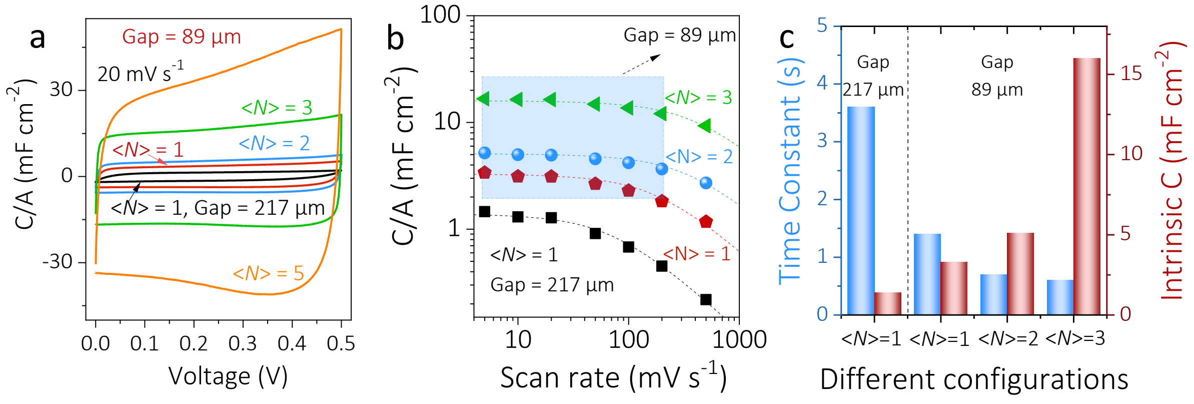

Supplementary Figure 22. Electrochemical characterizations of MSCs with different printing pass. **a**, CV and **b**, areal capacitance curves of extrusion-printed MXene MSCs (line gap of 89 µm) at different scan rates. The data of MSC with line gap of 217 µm is also included for comparison. **c**, the areal capacitance of different MSCs with different <*N*> and line gaps.

As shown in Figure S22c, by increasing the printing path, a higher areal capacitance is achieved. In addition, the line gap turns out to be a critical parameter that strongly influences both, the achievable areal capacitance (that is, intrinsic areal capacitance, C_A) and the time constant (τ). These two parameters can be obtained by fitting the calculated areal capacitance according to the empirical equation:[8,9]

$$C/A = C_A[1 - \frac{v\tau}{\Delta V}(1 - e^{-\frac{\Delta V}{v\tau}})]$$

where ΔV is the voltage window (0.5 V), v is the scan rate. By plotting C_A and τ as a function of $<N>$, as shown in Figure S22c, it becomes clear that a larger line gap (217 μm) limits the ion diffusion kinetics, while C_A increases as $<N>$. The sharp increase of C_A suggests a well percolated network is formed when depositing three paths of MXene ink.[1,10]

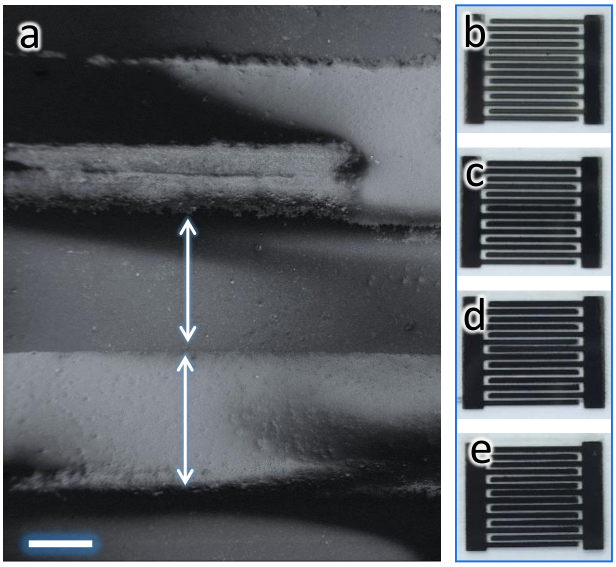

Supplementary Figure 23. Morphological study of various MSCs printed using different solvents. **a**, SEM of inkjet-printed MSC using NMP ink. Optical images of inkjet-printed MSCs using **b**, ethanol, **c**, DMSO, **d**, DMF and **e**, NMP inks, respectively. The printed line width is ~450 μm and line gap ~410 μm in all the MSCs. Scale bar is 200 μm. The distance along the arrow is ~410 μm (top) and ~450 μm (bottom).

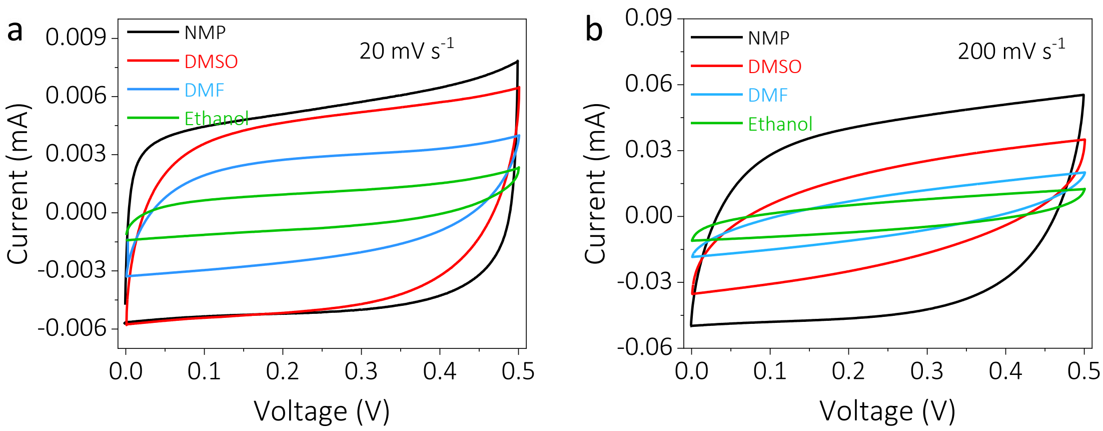

Supplementary Figure 24. CVs of MXene MSCs. CV curves comparison of the inkjet-printed all-MXene MSCs at **a**, 20 mV s^{-1} and **b**, 200 mV s^{-1}. The quasi-rectangular CV shape at low rates in all MSCs suggests the capacitive behaviour. Moreover, it shows NMP ink-based MSC demonstrated the best electrochemical performance.

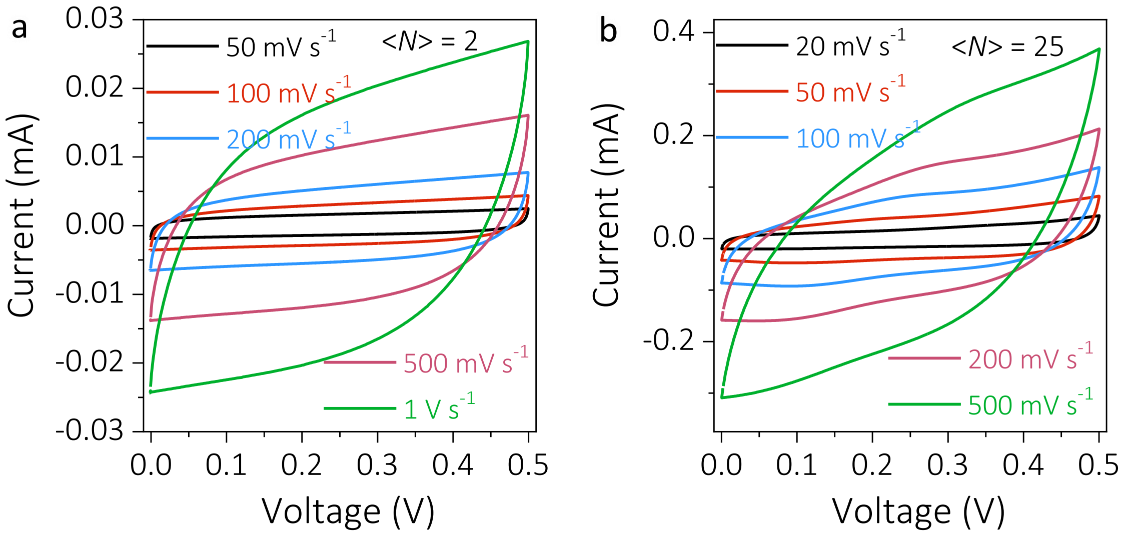

Supplementary Figure 25. CVs of MXene MSCs with different printing pass. CV curves of inkjet-printed MXene MSCs with **a**, <*N*>=2 and **b**, <*N*>=25 using NMP ink.

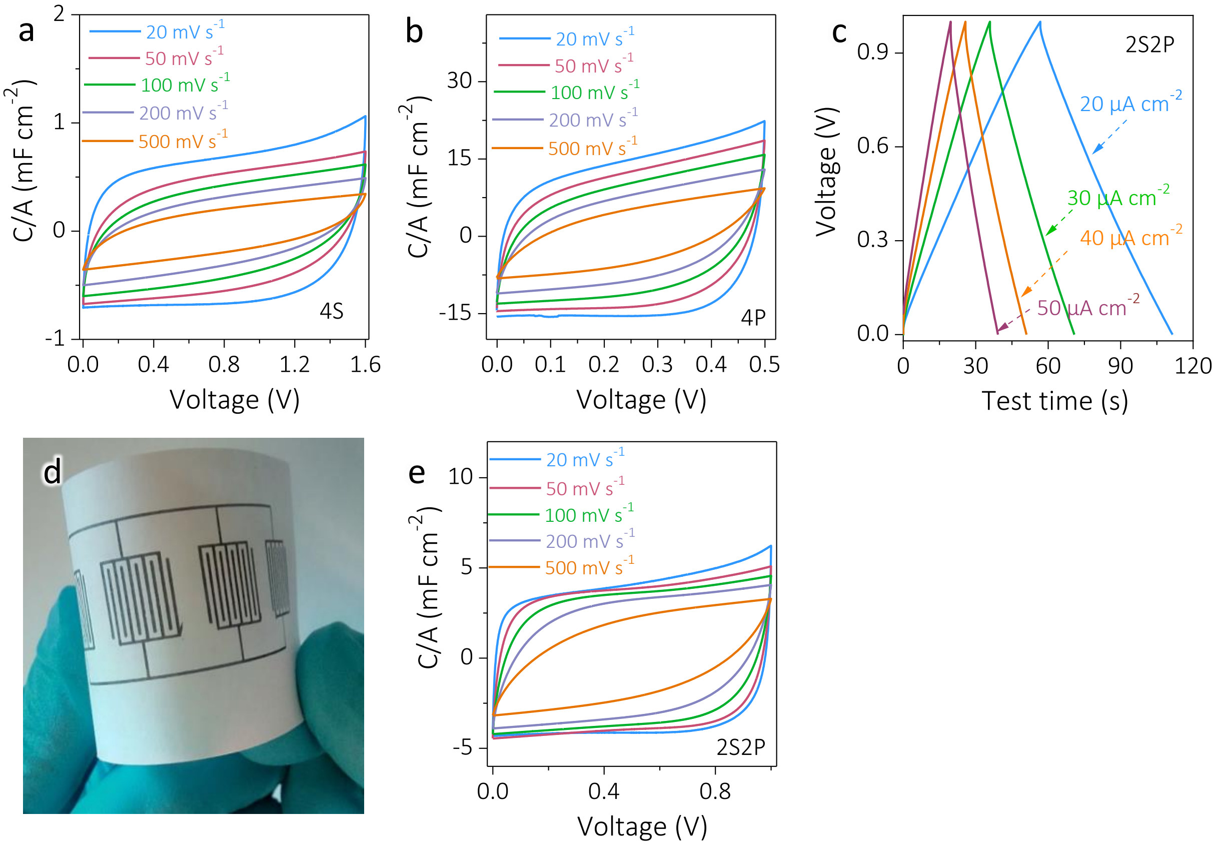

Supplementary Figure 26. Electrochemical characterization of tandem devices based on extrusion-printed MXene MSCs. CV curves of **a**, 4 MSCs printed in series (4S), **b**, 4 MSCs printed in parallel (4P). **c**, GCD curves and **d**, Optical image of 4P device, showing a great flexibility. **e**, CV curves of 2S2P tandem device.

By printing four MSCs in series, the tandem device reversibly stores the charge in a voltage window of 1.6 V. By printing two MSCs in series and then connecting another 2S device in parallel, forming 2S2P configuration, the tandem device can be tested in a voltage window of

1.0 V with twice as large an output current. All CVs and GCD profiles show good capacitive behaviour in these tandem devices, including rectangular CV shape and symmetric, linear GCD lines with negligible IR drop, suggesting the all-MXene-printed MSCs hold great promise for satisfying the energy/power demands for a variety of flexible electronics applications.

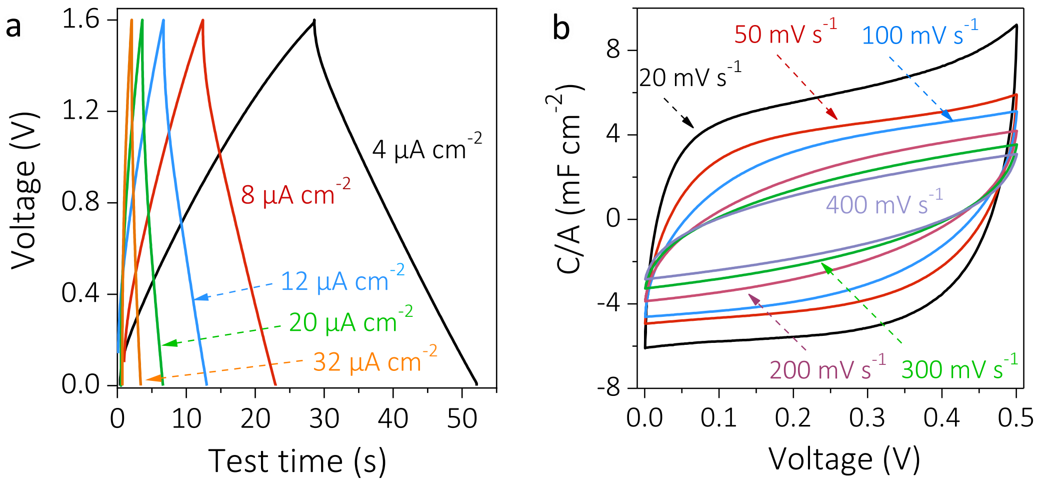

Supplementary Figure 27. Electrochemical characterization of MXene tandem device. **a**, GCD curves of tandem device by inkjet printing 4 MSCs in series, demonstrating a stable voltage window of 1.6 V with negligible IR drop, indicative of reversible charge storage. **b**, Normalized CV curves of inkjet-printed 4P tandem device, showing a capacitive behaviour as expected.

Supplementary Table

Supplementary Table 1. Fluidic properties of MXene organic inks

Organic MXene ink	Concentration (mg mL^{-1})	Density (g cm^{-3})	Viscosity (mPa·s)	Surface tension (mN m^{-1})	Ohnesorge number, *Z*
Ethanol	0.8	0.791	7.3	22.1	2.6
NMP	12.5	1.05	13.8	41.0	2.2
DMSO	2.1	1.102	12.8	43.5	2.5

Note: Fluidic properties of MXene-DMF ink is not possible due to lab restrictions.

Supplementary Table 2. Areal capacitance comparison of various MSCs (Fig. 5e)

Material	MSC fabrication method	Areal capacitance per electrode (C/A, mF cm^{-2})	Reference
Graphene	Inkjet printing	0.068	11
Graphene	Coating-masking-etching	0.1	12
PEDOT/MnO_2	Inkjet printing	0.26	13
Graphene	Self-aligned printing	0.27	14
Electrochemical exfoliated graphene	Inkjet printing	0.7	15
Electrochemical exfoliated graphene	Spray coating	0.8	16
Graphene	Inkjet printing	0.82	17
Graphene/PEDOT (G-PEDOT)	Spray coating	1.0	18
Graphene/MXene (G-MX)	Spray coating	3.2	19
Graphene/PEDOT (G-PEDOT)	Spray coating	5.4	16
Graphene	Layer-by-layer printing	19.8	20
$Ti_3C_2T_x$ MXene	Laser scribing	24.8	21
$Ti_3C_2T_x$ MXene	Laser cutting	27	22
$Ti_3C_2T_x$ MXene	Inkjet printing	12	This work
$Ti_3C_2T_x$ MXene	Extrusion printing	43	This work

Supplementary Table 3. Volumetric capacitance comparison of various MSCs (Fig. 5f)

Material	MSC fabrication method	Volumetric capacitance per electrode (C/V, F cm^{-3})	Reference
Onion-like carbon (OLC)	Electrophoretic deposition (EPD)	5.2	23
graphene	Self-aligned printing	7	14
PEDOT/MnO_2	Inkjet printing	9.6	13
Graphene	DVD laser scribing	12.2	24
GO/PANi/PEDOT (G-PA-PEDOT)	Extrusion printing	19.2	25
Graphene/PEDOT (G-PEDOT)	Spray coating	27	16
Graphene/MXene (G-MX)	Spray-masking	33	19
Graphene	Inkjet printing	37.2	11
Electrochemical exfoliated graphene	Inkjet printing	93.3	15
$Ti_3C_2T_x$ MXene	Inkjet printing	562	This work

Supplementary Table 4. Energy density & power density comparison of various MSCs (Fig. 5g)

Sample	Power density (μW cm^{-2})	Energy density (μWh cm^{-2})	reference
Reduced graphene oxide (rGO)	9	0.014	26
Graphene quantum dots (GQD)	7.5	0.074	27
PEDOT/Ag	138	0.041	21
	575	0.04	
	1480	0.034	
	2290	0.022	
	2880	0.019	
Inkjet-printed graphene (IJP-graphene)	0.025	0.00139	12
	0.045	0.00125	
	0.1	0.00111	
	0.175	9.72E-04	
	0.3	8.33E-04	
Spray-coated graphene/PEDOT (G-PEDOT)	0.8	0.08889	19
	1.6	0.07111	
	3	0.06667	
	5.6	0.06222	
	12	0.05333	
	20.8	0.04622	
	40	0.04444	
Spray-coated graphene (S-graphene)	0.2	0.02778	19
	0.40625	0.02257	
	0.7875	0.02187	
	1.35	0.01875	
	2.8125	0.01563	
	4	0.01111	
	5	0.00694	
Graphene quantum dots/MnO_2 (GQD- MnO_2)	7.5	0.154	27
Extrusion-printed all-MXene MSC	11.4	0.3166	This work
	22.5	0.3120	
	44.1	0.3065	
	75.1	0.2086	
	115.4	0.1603	
	157.7	0.1094	

Supplementary Note

Preparation of multi-layer $Ti_3C_2T_x$ MXene "cake". 0.5 g of lithium fluoride (LiF, Sigma Aldrich, USA) was added to 10 mL of 9 M hydrochloric acid (HCl, 37 wt%, Sigma Aldrich, USA) under stirring at room temperature until a clear solution was formed. Then, 0.5 g of Ti_3AlC_2 MAX phase (average particle size ~38 µm, Y-Carbon, Ukraine) was slowly added to the above mixture over the course of 40 min under vigorous stirring, then transferred to an oil bath and reacted for 24 h at 35 °C at a stirring speed of 200 rpm. Once the reaction ended, the suspension was transferred to centrifuge tubes and centrifuged at 3500 rpm for 3 min. The supernatant was then decanted, and 35 mL of fresh DI water was added to the suspension followed by vigorous manual shaking for 1 min, then subjected to another round of centrifugation at 3500 rpm for 3 min. This washing process was repeated 4 times until the pH of the supernatant became ~6. After decanting the supernatant, the multi-layered (m-) $Ti_3C_2T_x$ sediment was collected and filtrated through a membrane (Celgard 3501, Celgard, USA). After being dried in ambient conditions, the entire specimen was transferred to the fridge and denoted as m-$Ti_3C_2T_x$ "cake".

Rheological characterization. The rheological behaviour of $Ti_3C_2T_x$ organic inks (NMP, DMSO, and ethanol) was studied on an Anton Paar MCR 301 rheometer using a PP50, parallel plate geometry (diameter of 50 mm, gap of 0.5 mm), with the exception of high concentration measurements, which were performed on a PP25, parallel plate geometry (diameter of 25 mm, gap of 0.5 mm) due to increased sample viscosity and reduced sample volume. A solvent trap was employed for all measurements to minimize solvent evaporation during the measurement. All the loaded samples were allowed to equilibrate at room temperature for 1 minute. Oscillatory strain sweeps were performed first at a frequency of 0.5 Hz, from 0.5-100% strain, with consecutive measurements performed to ensure reproducibility. Apparent viscosities of the organic inks were measured *via* steady-state continuous shear experiments with a sweep of shear rate (5-100 s^{-1}).

Materials characterization. Delaminated MXene nanosheets were imaged by transmission electron microscopy (TEM, FEI Titan, USA) operated at 300 kV. TEM samples were prepared by diluting the organic inks and then drop-casting onto ultra-thin holey carbon copper grids (400 mesh - Agar). The lateral size histogram of the nanosheets was obtained by measuring sufficient numbers of flakes (N >150) from TEM images. Energy-dispersive X-ray spectroscopy (EDX) was acquired using the EDAX detector fitted on the FEI Titan. Scanning electron microscopy (SEM) images of the printed lines/patterns were acquired on a Zeiss Ultra Plus (Carl Zeiss, Germany) with an acceleration voltage of 2 keV. The width of printed lines was measured along the axial direction from the SEM images. The spatial uniformity was obtained from the width variation statistics.

Height profiles of the as-printed lines were acquired under ambient conditions using a Bruker Multimode 8 system in peak force tapping mode, with Nanosensors PPP-NCLR probes with a tip resonant frequency of 190 kHz. The data was processed with Nanoscope analysis and Gwyddion software. A silicon wafer was used as the substrate for the nanosheet-AFM measurements while the as-printed lines were directly measured on the AlO_x-coated PET without further modification. It is worth noting that the roughness of the printed lines could be considered due to the rough surface from the PET substrate.

Raman spectra of vacuum-filtrated films made of delaminated nanosheets, as well as the inkjet and extrusion-printed lines, were acquired on a WITec Alpha 300 R confocal Raman microscope. The excitation wavelength was 532 nm and the laser power ~ 300 µW.

XPS spectra were obtained using monochromated Al Kα X-rays from an Omicron XM1000 MkII X-ray source. An Omicron EA125 energy analyzer was used with pass energy set to 20 eV for the core-level spectra, which were then deconvoluted and fitted using the CasaXPS software. The data was charge corrected to the C 1s at 248.8 eV and after subtraction of the background (70% Shirley, 30% linear) was fitted giving deconvoluted peaks.

The sheet resistance of the as-printed lines was measured using a four-point probe method on a Keithley 2400 source meter. Ag paint was applied on the stripes and four Ag wires were attached to the paint for cable connections. The sheet resistance of the lines with different passes was converted to the electrical conductivity based on the line length, width and thickness. A bias voltage was applied on the inkjet-printed lines using a potentiostat (VMP3, BioLogic, France) at a sweep rate of 20 V s^{-1}. Currents were recorded and *I-V* curves were obtained. The resistance of these as-printed lines was measured (using a multimeter) as a function of line length (gap between two painted Ag contact), which was measured under an optical microscope.

To study the robustness of the printed lines, the inkjet- and extrusion-printed samples with AlO_x-coated PET and paper as the substrate, respectively, were cut into strips. The two ends of the strips were fixed to the substrates. Two lines of Ag paint were brushed onto the two ends of the strip, which was connected to a multimeter through two pieces of Ag wire. A piece of scotch tape was used to cover/fasten the Ag paints to the moving plates, ensuring no cracking on the Ag paints, as well as the connecting point. The resistances of the strips upon bending at different degrees were recorded and converted to the electrical conductivity. The conductivity was further plotted as a function of bending degree. A complete bending cycle was defined as 0°→ 180°→ 0°. For the cycling tests, the strips were bent for 1000 cycles and the resistance was recorded accordingly.

Solid-state, on-chip MSC fabrication: Since $Ti_3C_2T_x$ MXene exhibits a metallic conductivity, and thus behaves as both the active material and current collector for supercapacitors, no extra metal current collector was involved. To facilitate the measurements, two Ag wires were attached at the end of the MSC, and were glued by Ag paint. After 10 min drying, two lines of nail polish were coated on top of the Ag paint to protect the latter from potential exposure to the electrolyte. After that, both inkjet- and extrusion-printed MSCs were coated with a layer of gel-like polymer electrolyte made of 3M sulfuric acid (H_2SO_4)-Poly(vinyl alcohol, PVA) gel electrolyte. The preparation of gel electrolyte can be found in ref 1. After naturally drying, solid-state, on-chip MSCs were formed, which were stored under vacuum for further measurements.

Electrochemical characterization: The electrochemical performance of the as-fabricated MSCs was evaluated through CV and GCD on a VMP3 potentiostat (BioLogic, France). The devices were tested at different CV scan rates (from 10 to 1000 mV s^{-1}) and GCD current densities (from 2 to 200 µA cm^{-2}) in a voltage window of 0.5 V. The cycling performance of the devices was evaluated at 14 µA cm^{-2} for inkjet-printed MSCs and at 200 µA cm^{-2} for extrusion-printed ones. Electrochemical impedance spectroscopy was performed at the open-circuit voltage from 100 mHz to 100 kHz. The areal capacitance per electrode was calculated from the fifth CV curve according to the following equation:

$$C/A = \frac{4}{A_t \Delta V v} \int_0^{0.6} j dV \qquad (1)$$

Where, *C/A* is the measured areal capacitance (mF cm^{-2}), j is the current (mA), ΔV is the voltage window (0.5 V), A_t (cm^2) is the geometric area of the MSC, v is the scan rate (mV s^{-1}).

In the devices that showcased symmetric, linear GCD curves, the areal capacitance was also obtained from the fifth GCD curves according to the following equation:

$$C/A = \frac{4j\Delta t}{A_t \Delta V} \tag{2}$$

where ΔV is the effective voltage window after the IR drop, Δ*t* is the discharge time.

The volumetric capacitance per electrode in the inkjet-printed MSCs was also calculated based on the following equation:

$$C/V = \frac{C/A}{t} \tag{3}$$

where *t* is the film thickness of the printed lines (measured by AFM).

For the Ragone plot, the device areal energy density and power density were calculated based on the following equations:

$$\frac{C_D}{A} = \frac{\int_0^{0.6} j dV}{A_t \Delta V \upsilon} \tag{4}$$

$$\frac{E_D}{A} = \frac{C_D \times V^2}{A \times 2 \times 3.6} \tag{5}$$

$$\frac{P_D}{A} = \frac{E_D \times \nu \times 3600}{A \times \Delta V} \tag{6}$$

where C_D/A (mF cm^{-2}) is the areal capacitance of the device, E_D/A is the device areal energy density (µWh cm^{-2}), P_D/A is the device areal power density (µW cm^{-2}), *j* is the current (mA), ΔV is the voltage window (0.5 V), A_t (cm^2) is the geometric area of the MSC, υ is the scan rate (mV s^{-1}).

Supplementary References

1. Zhang, C. J. *et al.* Stamping of Flexible, Coplanar Micro-Supercapacitors Using MXene Inks. *Adv. Funct. Mater.* **28,** 1705506 (2018).
2. Ghidiu, M., Lukatskaya, M. R., Zhao, M.-Q., Gogotsi, Y. & Barsoum, M. W. Conductive two-dimensional titanium carbide 'clay' with high volumetric capacitance. *Nature* **516,** 78–81 (2014).
3. Barwich, S., Coleman, J. N. & Möbius, M. E. Yielding and flow of highly concentrated, few-layer graphene suspensions. *Soft Matter* **11,** 3159–3164 (2015).
4. Zhang, C. J. *et al.* Oxidation Stability of Colloidal Two-Dimensional Titanium Carbides (MXenes). *Chem. Mater.* **29,** 4848–4856 (2017).
5. Sun, H. *et al.* Binder-free graphene as an advanced anode for lithium batteries. *J. Mater. Chem. A* **4,** 6886–6895 (2016).
6. Charlier, J., Cousty, J., Xie, Z. X., Poulennec, C. V.-L. & Bureau, C. Adsorption of substituted pyrrolidone molecules on Au(111): an STM and XPS study. *Surf. Interface Anal.* **30,** 283–287 (2000).
7. Peng, H. *et al.* Effect of Transition Metals on the Structure and Performance of the Doped Carbon Catalysts Derived From Polyaniline and Melamine for ORR Application. *ACS Catal.* **4,** 3797–3805 (2014).
8. Zhang, C. *et al.* Highly flexible and transparent solid-state supercapacitors based on RuO_2/PEDOT:PSS conductive ultrathin films. *Nano Energy* **28,** 495–505 (2016).
9. Higgins, T. M. & Coleman, J. N. Avoiding Resistance Limitations in High-Performance Transparent Supercapacitor Electrodes Based on Large-Area, High-Conductivity PEDOT:PSS

Films. *ACS Appl. Mater. Interfaces* **7,** 16495–16506 (2015).
10. King, P. J., Higgins, T. M., De, S., Nicoloso, N. & Coleman, J. N. Percolation effects in supercapacitors with thin, transparent carbon nanotube electrodes. *ACS Nano* **6,** 1732–1741 (2012).
11. Li, L. *et al.* High-Performance Solid-State Supercapacitors and Microsupercapacitors Derived from Printable Graphene Inks. *Adv. Energy Mater.* **6,** 1600909 (2016).
12. Sollami Delekta, S., Smith, A. D., Li, J. & Östling, M. Inkjet printed highly transparent and flexible graphene micro-supercapacitors. *Nanoscale* **9,** 6998–7005 (2017).
13. Wang, Y., Zhang, Y.-Z., Dubbink, D. & ten Elshof, J. E. Inkjet printing of δ-MnO_2 nanosheets for flexible solid-state micro-supercapacitor. *Nano Energy* **49,** 481–488 (2018).
14. Hyun, W. J. *et al.* Scalable, Self-Aligned Printing of Flexible Graphene Micro-Supercapacitors. *Adv. Energy Mater.* **7,** 1700285 (2017).
15. Li, J. *et al.* Scalable Fabrication and Integration of Graphene Microsupercapacitors through Full Inkjet Printing. *ACS Nano* **11,** 8249–8256 (2017).
16. Liu, Z. *et al.* Ultraflexible In-Plane Micro-Supercapacitors by Direct Printing of Solution-Processable Electrochemically Exfoliated Graphene. *Adv. Mater.* **28,** 2217–2222 (2016).
17. Li, J. *et al.* Efficient Inkjet Printing of Graphene. *Adv. Mater.* **25,** 3985–3992 (2013).
18. Wu, Z.-S., Liu, Z., Parvez, K., Feng, X. & Müllen, K. Ultrathin Printable Graphene Supercapacitors with AC Line-Filtering Performance. *Adv. Mater.* **27,** 3669–3675 (2015).
19. Li, H. *et al.* Flexible All-Solid-State Supercapacitors with High Volumetric Capacitances Boosted by Solution Processable MXene and Electrochemically Exfoliated Graphene. *Adv. Energy Mater.* **7,** 1601847 (2017).
20. Sun, G. *et al.* Layer-by-layer printing of laminated graphene-based interdigitated microelectrodes for flexible planar micro-supercapacitors. *Electrochem. commun.* **51,** 33–36 (2015).
21. Kurra, N., Ahmed, B., Gogotsi, Y. & Alshareef, H. N. MXene-on-Paper Coplanar Microsupercapacitors. *Adv. Energy Mater.* **6,** 1601372 (2016).
22. Peng, Y.-Y. *et al.* All-MXene (2D titanium carbide) solid-state microsupercapacitors for on-chip energy storage. *Energy Environ. Sci.* **9,** 2847–2854 (2016).
23. Pech, D. *et al.* Ultrahigh-power micrometre-sized supercapacitors based on onion-like carbon. *Nat. Nanotechnol.* **5,** 651 (2010).
24. El-Kady, M. F. & Kaner, R. B. Scalable fabrication of high-power graphene micro-supercapacitors for flexible and on-chip energy storage. *Nat. Commun.* **4,** 1475 (2013).
25. Liu, Y. *et al.* Development of Graphene Oxide/Polyaniline Inks for High Performance Flexible Microsupercapacitors via Extrusion Printing. *Adv. Funct. Mater.* **28,** 1706592 (2018).
26. Yoo, J. J. *et al.* Ultrathin planar graphene supercapacitors. *Nano Lett.* **11,** 1423–1427 (2011).
27. Liu, W.-W., Feng, Y.-Q., Yan, X.-B., Chen, J.-T. & Xue, Q.-J. Superior Micro-Supercapacitors Based on Graphene Quantum Dots. *Adv. Funct. Mater.* **23,** 4111–4122 (2013).
